# Spatio-temporal and -spectral feature maps in photoplethysmography imaging and infrared thermograph

**DOI:** 10.1186/s12938-020-00841-9

**Published:** 2021-01-07

**Authors:** Michael Paul, Sabrina Caprice Behr, Christoph Weiss, Konrad Heimann, Thorsten Orlikowsky, Steffen Leonhardt

**Affiliations:** 1 Medical Information Technology (MedIT), Helmholtz-Institute for Biomedical Engineering, RWTH Aachen University, Pauwelsstr. 20, 52074 Aachen, Germany; 2 Uniklinik RWTH Aachen, Section of Neonatology, Pauwelsstr. 30, 52074 Aachen, Germany

**Keywords:** Photoplethysmography imaging, Infrared thermography, Camera-based, Imaging, Remote, Non-contact, Spatio-temporal, Spatio-spectral

## Abstract

**Background:**

Only a small fraction of the information available is generally used in the majority of camera-based sensing approaches for vital sign monitoring. Dedicated skin pixels, for example, fall into this category while other regions are often disregarded early in the processing chain.

**Methods:**

We look at a simple processing chain for imaging where a video stream is converted to several other streams to investigate whether other image regions should also be considered. These streams are generated by mapping spatio-temporal and -spectral features of video segments and, thus, compressing the information contained in several seconds of video and encoding these in a new image. Two typical scenarios are provided as examples to study the applicability of these maps: face videos in a laboratory setting and measurements of a baby in the neonatal intensive care unit. Each measurement consists of the synchronous recording of photoplethysmography imaging (PPGI) and infrared thermography (IRT). We report the results of a visual inspection of those maps, evaluate the root mean square (RMS) contrast of foreground and background regions, and use histogram intersections as a tool for similarity measurements.

**Results:**

The maps allow us to distinguish visually between pulsatile foreground objects and an image background, which is found to be a noisy pattern. Distortions in the maps could be localized and the origin could be discovered. The IRT highlights subject contours for the heart frequency band, while silhouettes show strong signals in PPGI. Reflections and shadows were found to be sources of signals and distortions. We can testify advantages for the use of near-infrared light for PPGI. Furthermore, a difference in RMS contrast for pulsatile and non-pulsatile regions could be demonstrated. Histogram intersections allowed us to differentiate between the background and foreground.

**Conclusions:**

We introduced new maps for the two sensing modalities and presented an overview for three different wavelength ranges. The maps can be used as a tool for visualizing aspects of the dynamic information hidden in video streams without automation. We propose focusing on an indirect method to detect pulsatile regions by using the noisy background pattern characteristic, for example, based on the histogram approach introduced.

## Background

### Motivation

Camera-based sensing of vital parameters is a recent research topic in many application fields, such as clinical monitoring, driver-state estimation, emotion detection and even anti-spoofing in biometrics [1]. Most of the time, conventional video cameras are used for the task because they are affordable, widely available and often return color data, which humans are used to. In addition, cameras sensitive to other parts of the electromagnetic spectrum are also used, for example, thermal cameras, which are more and more used for fever screening at airports.

Nevertheless, there is neither a standardized measurement setup nor an agreement on algorithms even for conventional cameras [2]. This is not surprising considering the many different areas of application. However, standard implementations of certain algorithms have been available since 2019 [3–5]. Moreover, one other aspect is the reduction of data in an early processing stage to maintain manageability, especially when facing videos that are both very bandwidth- and storage-intense. It is known that lossy, conventional video compression algorithms deteriorate the useful signal [6]. Consequently, a first approach was presented to overcome this problem [7] but is not widely used. Thus, it is no surprise that when extracting dynamic information for vital sign sensing for real-time applications, videos are often reduced to image regions. These regions of interest (ROIs) might contain only the patient or a small portion of the skin (e.g., face, hand [8]). However, the surrounding area is often not considered at the expense of discarding information which might be useful in signal retrieval and subsequent processing steps (e.g., artifact removal). A few examples of what can happen outside the ROIs are as follows: the movement of caregivers in a clinical monitoring scenario affects the lighting conditions, for example, by casting unwanted shadows, medical devices emit light signals influencing the scene, or tubes and wires are moving (partly following breathing movements of the patient). While the presence of the first two introduces artifacts, the occurrence of movement coupled to body activity might contain the vital sign anticipated.

Hence, we will look in the following at the regions not yet exploited. Dynamic components of video segments can be compressed to 2D images for this purpose.

### State of the art

The two sensing modalities photoplethysmography imaging (PPGI) and infrared thermography (IRT) were used in this work.

PPGI is known by various names [9, 10] and can be used to extract the PPG signal (changes in light intensity that are modulated by blood volume pulsations) and ballistographic signals (signals resulting from movement, for example, from blood ejected by the heart [11]) remotely. The IRT, or thermal imaging [12], uses specialized cameras sensitive to thermal radiation emitted by objects and subjects. One of its prevalent medical applications is the screening of fevers (e.g., at airports), but also the extraction of vital signs (predominantly breathing by exploiting temperature differences at the nostrils or by detecting movements of the chest). One other aspect in which the modalities differ is spatial resolution: PPGI can be used with cameras, both charge-coupled devices (CCD) and complementary metal-oxide semiconductors (CMOS), which are mass-market products and, nowadays, typically have high-definition (HD) resolutions (above $1280 \times 720\hbox { pix}$) at a reasonably low price. By contrast, IRT cameras use special sensors (e.g., bolometers) typically equipped with $640 \times 480\hbox { pix}$ (or below) because higher resolving sensors are still very expensive (several thousand dollars). However, we have witnessed a price drop in this area in recent years and the first low-cost devices have entered the market.

The typical signal extraction for video processing is described as follows: depending on the application, the first step is to detect the subject, and the second step involves locating one or multiple ROIs which are then tracked over time. Pixels of a ROI can be pooled (often by spatial averaging) and, thus, a time series is created. Signals of different ROIs (or different optical wavelengths in the case of PPGI) can be optionally combined to form a signal which is finally analyzed by one-dimensional signal processing.

Algorithms can rely on a separate reference ROIs which do not contain the signal to improve the signal extracted. Such an approach that employs autoregressive modeling was developed by Tarassenko and co-workers to compensate for the effects, for example, of artificial light flicker in the visible light range [13]. However, the authors did not state how to choose this reference ROI, but define it manually on an empirical basis.

Furthermore, the spatial resolution was exploited by several groups in various ways: Kamshilin and co-workers [14] used synchronous detection and applied lock-in amplification on every pixel of a video stream to make blood volume pulsations more visible. A reference signal required for the approach was obtained from a reference ROI of the same video stream. Interestingly, this approach may be applied to any frequency band of interest. It was shown that not only the amplitude, but also the phase of the blood volume pulsations is unevenly distributed [15].

Bobbia and co-authors [16] pointed out that the identification of a well-defined ROI (number of skin pixels and temporal stability) is not a simple task. Instead of relying on direct skin segmentation (e.g., based on color or body part detectors), the authors relied on the assumption that living skin exhibits pulsatility, while non-skin pixels would not. Thus, in order to identify pulsatile regions, the whole image was unequally divided by a superpixel approach (which clusters pixels of similar properties) resulting in non-rectangular ROIs.

Amelard and co-workers [17] developed a spatial probabilistic pulsatility model that allowed them to identify regions of strong pulsatility for their specific scenario. Such a model-based approach could be adapted to different scenarios. This would also allow the generation of new models for different body parts, views, illumination, wavelengths, etc.

The Eulerian video magnification approach [18] magnifies the dynamic information in video sequences, such as blood pulsations, and makes these more visible in amplified video sequences. This process involves spatially low-pass filtering of the images and downsampling for computational efficiency, and, generally contains a full Laplacian pyramid of different spatial resolutions. Dynamic information in Eulerian video magnification is considered by temporal bandpass filtering of pixel series. Hence, the approach is useful for the visualization of dynamic processes.

Approaches exploiting deep learning have emerged more recently. One example is the work of Bousefsaf et al. [19] where the team uses 3D convolutional neural networks to estimate the pulse rate. Their approach is to generate pulse prediction maps where frequencies are mapped to pixel positions. They developed a network that operates on small spatio-temporal video patches for this.

**Fig. 1:**
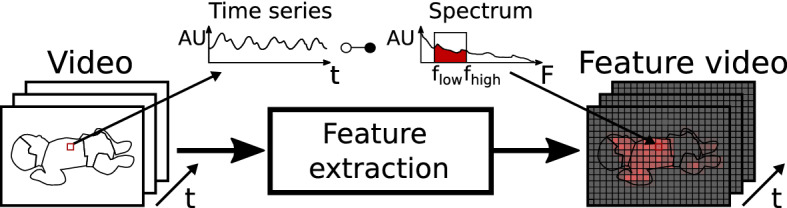
Feature video generation. Dynamic information of a video segment is compressed to images. Processing the whole video yields new video streams. The same processing is used for each image region on a grid (‘cell’)

In fact, shortly after camera-based sensing had been used to extract signals from single or multiple ROIs, imaging was researched, which involves at least the spatial pooling of pixels to form ROIs and subsequent temporal filtering. These approaches use ROIs laid out on a grid on the image (sometimes, the ROIs are referred to as cells). An image is obtained by extracting a signal for each ROI and mapping a signal characteristic (feature) to the ROIs’ position (see Fig. 1). This is typically the PPG amplitude (e.g., [8, 20]) but can also include the blood pulse phase and other waveform parameters [1, 15, 21–23]. The approaches of Verkruysse et al. and Kumar et al. are particularly noteworthy for this paper: Verkruysse and co-workers [21] defined and generated power maps and phase maps at single frequencies, i.e., they analyzed the Fourier spectrum at the known heart frequency and mapped the signal power. Kumar et al. [24] combined a cell-based approach with tracking to extract a more robust pulse signal. They calculated spatio-spectral maps for this which are based on the power spectral density. They used the latter to define a mapping of a goodness metric, which is essentially the power in a band close to an initial pulse rate estimate versus the remaining power of the signal. The power spectral density was also exploited by Fallet and co-workers [25] who used the power in the band close to the heart rate known to determine suitable ROIs in the face for signal retrieval. Other approaches exploit motion in grid cells to estimate breathing activity [26, 27].

Furthermore, it is also possible to extract information about movement without calculating motion vectors: We used difference images to calculate movement maps that, for example, can help to identify good ROIs [28], i.e., those less affected by movement.

Cell-based approaches also exist for IRT: exemplarily, breathing was extracted in [29].

As has been described above, the conventional method of signal retrieval is to locate and track ROIs and extract signals. An approach that does not rely on tracking but requires multiple color channels was presented in [30].

An application besides vital sign monitoring is anti-spoofing in biometrics, where real faces need to be discerned from fake faces (masks, photos). Heusch et al. [31] applied PPGI for this and used spectral statistics (first- and second-order) derived from the pulse-signal for face presentation attack detection.

### Approach

In this work, spatio-temporal and -spectral features are extracted from a relatively dense grid of evenly spaced ROIs and mapped to new feature videos. Similar to Bobbia et al. [16], we exploit the fact that the signals have a certain temporal characteristic (i.e., pulsatility). However, we do not look at a complete processing chain for vital sign retrieval. Instead, we focus on the presence of the pulsatile image regions and the usefulness of spatio-temporal and -spectral maps.

According to [22], this usefulness was not always ascertainable in previous works. We are facing and objecting this criticism by introducing various new maps.

This paper deals with the aspects of choosing good regions by exploring whether the foreground (pulsatile image segments) are highlighted against a less ordered/noisy background and if we can determine the noise sources.

As stated above, the precise identification of ROIs is a non-trivial task. Moreover, it is interesting to see whether only skin pixels can contribute to useful signals. That is why we aim at the extraction of dynamic information, such as pulse or breathing rate, from video sequences. We will present and discuss maps of two subjects regarding signals in the heart frequency band to give examples.

Let us assume, for this, that the content of the video is unknown. Moreover, we want to extract the signals from sources that can have arbitrary shapes. In addition, we want to explore which image regions contain valuable information. Hence, we can rely neither on detectors for the skin, the pose nor body parts. Furthermore, we concentrate on monochrome data, which means that there is no color. Instead, only (light/radiation) intensity values are available. This is a useful constraint to apply to IRT generally or to PPGI scenarios where color information is not available, such as for example, at night using near-infrared (NIR) wavelengths. However, the approach can also be applied to visible light (VIS) which we will also cover.

We use tools for one-dimensional signal analysis to identify pulsatile signals. We rely on well-established methods based on Fourier analysis and signal statistics inter alia, particularly spectral descriptors, which are commonly used in speech and audio processing (e.g., [32, 33]). Our mapping approach is similar to the one used by Verkruysse et al. [21] to generate power maps. However, instead of generating maps for a particular single frequency (we do not know the frequency a priori), we introduce maps where each ROI is handled independently for a certain frequency range.

**Fig. 2:**
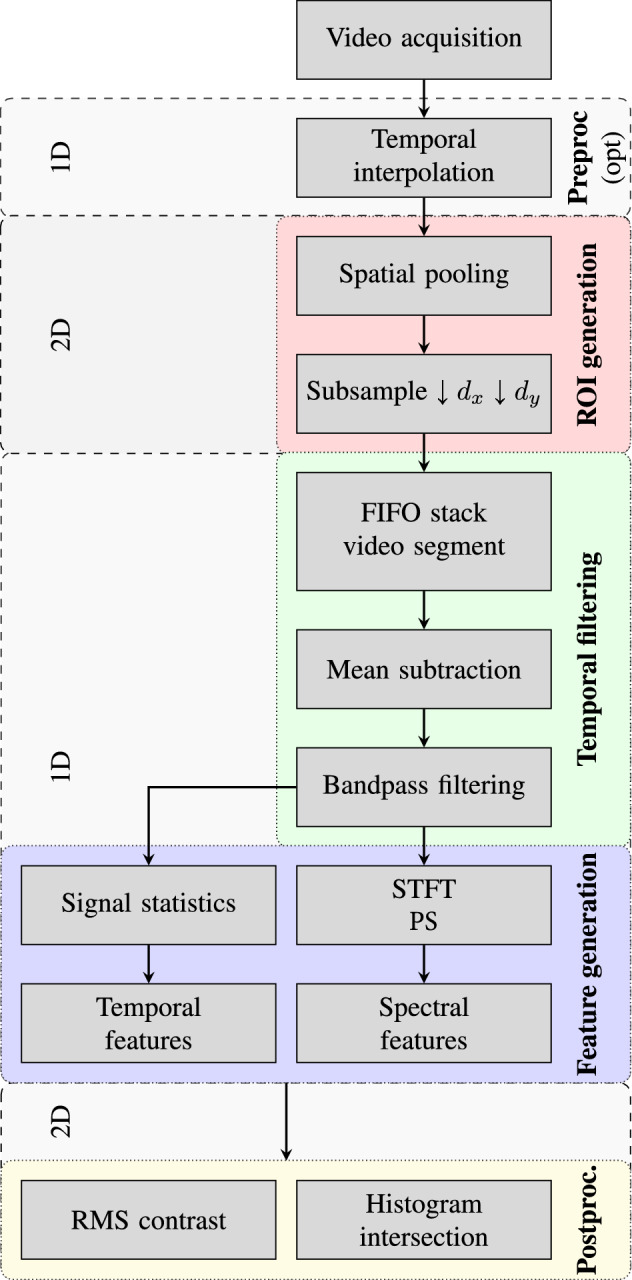
Video processing chain. Video segments pass through the three stages: ROI generation, temporal filtering and feature generation to create new feature maps. Firstly, pixels are spatially pooled. Images are then subsampled to a resolution which can be processed. Afterwards, pixel series which correspond to ROIs are processed. Consequently, the subsampled video segments are buffered in a first in, first out (FIFO) stack. Features are generated from short-time Fourier transforms (STFT), power spectra (PS) or signal statistics. An optional preprocessing adds artificial frames if there were losses during recording. We introduce a postprocessing for evaluating the maps: ‘Similarity maps’ are created by using histogram intersections and the root mean square (RMS) contrast is calculated for a manual selection of ROIs. The parametrization used in this work is given in Table 3 and the method is provided in Section "Methods" in detail

The basic idea is that each ROI can be treated as a one-dimensional signal (Fig. 1). The overview of the method for generating feature maps and the postprocessing steps for evaluation proposed are depicted in Fig. 2 and are presented in detail in Section "Methods". The ROIs are represented here by single pixels. Each ROI is constructed by spatially pooling pixels (blurring image patches). After subsampling, only a subset of the pixels pooled are further processed. Prior to calculating the features, the mean is subtracted from the pixel time series and these are filtered. Hence, dynamic components outside of a pre-defined frequency band are attenuated. Feature maps are generated by mapping the calculated features of the individual ROIs to the corresponding image positions. For the evaluation of the maps, we computed the image contrast of bigger, manually selected ROIs, which either present the background or a pulsatile region. Furthermore, the similarity between one of these ROIs (background) and small image patches is compared by using histogram intersections resulting in ‘similarity maps’.

The main contributions of this work are:

- The introduction of several new spatio-temporal and -spectral maps for the two sensing modalities PPGI and IRT,
- The identification of maps suitable for signal retrieval,
- Examples of the detection of noise sources,
- An algorithmic approach for discerning pulsatile and non-pulsatile image regions based on spatial histograms,
- and, consequently, recommendations for improving sensing setups.

### Outline

The remainder of this work is structured as follows: a selection of maps is displayed and described in Section "Results" and the results of the similarity measurements are presented. For this, short video sequences of an adult and of a baby with minimal body movements were processed and analyzed in the anticipated value range for the heart rate. We report our findings regarding movements, image detail and noise sources for three different wavelength ranges using PPGI and IRT. In Section "Discussion", we address the limitations of the RMS contrast and discuss the feature map approach presented regarding movements and different wavelengths. In addition, we encourage the use of the ‘similarity maps’ for segmentation. This section concludes with the implications for camera-based measurements. The key findings and future research directions are provided in Section "Conclusions and outlook". In Section "Methods", we describe the generation of the feature maps in detail and how these are evaluated using the RMS contrast and histogram intersections. The videos used for the evaluation and the measurement setup are presented. The mathematical descriptions of the features are provided in the appendix. These are supplemented by a detailed overview of the corresponding maps. Furthermore, we report the results for the sequences of the two subjects, which contain more intense body movement.

## Results

In this section, we present the results of our algorithmic approach for generating feature maps. The video sequences used consist of adult and baby measurements. One sequence without (w/o) and one with (w/) movement is analyzed for each subject (Figs. 11 and 12), as described in Section "Methods". Each measurement has been recorded synchronously in three wavelength ranges, i.e., in the VIS and NIR for PPGI and in the long-wave infrared (LWIR) for IRT. Ambient and measurement light (white and NIR light-emitting diodes) have been used to illuminate the subjects. The length of each sequence analyzed is 10 s.

Firstly, we present a selection of feature and similarity maps for the videos w/o movement. Subsequently, we look briefly at the results of the root mean square (RMS) contrast. We consider the same two ROIs for each feature map for this purpose: a region in the image background ($ROI_\text {BG}$) and a region that was expected to show pulsatility ($ROI_\text {PULSE}$). $ROI_\text {BG}$ is also used to compute the similarity maps. Those result from histogram intersections between $ROI_\text {BG}$ and local image patches (see Section "Methods").

### Maps

We generated 20 maps per video and wavelength (19 feature maps, one reference) and corresponding similarity maps for each sequence. However, we present only a selection in this part of the work, i.e., the *temporal variance*, *band mean power*, and *spectral flatness*. We use the *mean intensity* maps as visual references.

Moreover, we dedicate this part to the maps of the measurements w/o movement while the maps corresponding to the video segments w/ movement are given in the appendix for completeness. Thus, the complete overview of the feature and similarity maps is given in the appendix (Section C) ranging from Figs. 13, 14, 15, 16, 17, 18, 19 and 20 for the adult measurement and from Figs. 21, 22, 23, 24, 25, 26, 27 and 28 for the baby measurement.

#### Adult lab measurement w/o movement

**Fig. 3:**
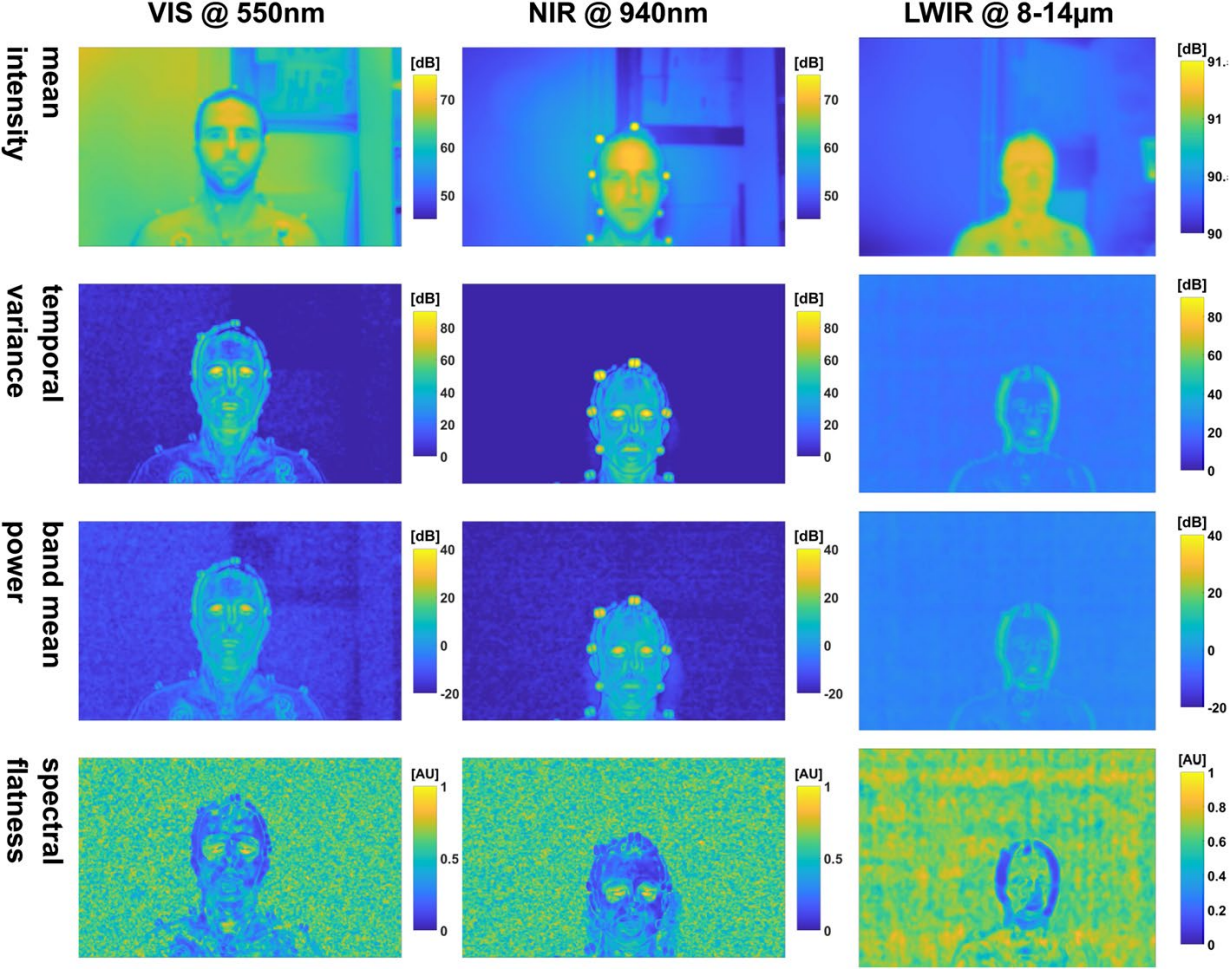
Selection of feature maps of the adult measurement w/o movement: *mean intensity* is used as a visual reference. All dynamic maps show a noisy background pattern. The maps *temporal variance* and *band mean power* look very similar. Both allow the identification of static background elements, hinting at non-constant illumination conditions. Regarding *spectral flatness*, the static background is not visible which could be advantageous for image segmentation

The maps for this video segment are given in Fig. 3 and in the appendix (Section C.1 Figs. 13 and  14).

The first thing to notice is that the IRT *mean intensity* image is the brightest, followed by the VIS and NIR PPGI camera. The simple reason is that the IRT camera uses the complete dynamic range, while the other cameras were not even close to being saturated. This is also true for the power (e.g., *band mean power*) in the frequency band of interest.

What can also be noticed is that PPGI and IRT enhance different image regions: when we look at the subject using PPG, the silhouette is visually enhanced, while for IRT, contours are emphasized. The head contour is highlighted the most, but also the torso contour. By contrast, the *mean intensity* image or the raw IRT image would allow an easy segmentation of the body silhouette.

Most PPGI maps allow easy identification of the head and facial components (the head is harder to discern only in the spectral *flux ratios*). It is easy to discriminate eyes, eyebrows, nose and mouth. It is even possible to identify the dark rings under the eyes. Facial components (especially the mouth) are also visible in IRT. Eyes and nose can be discerned by their contours and the regions below the eyes also show some dynamic behavior.

Small head movement has occurred, as can be spotted in PPGI, for example, at the reflective markers which were spatially smeared as a result.

As one would expect, we observed no uniform intensity distribution either on the subject or in the background (e.g., *band (mean) power)*. Moreover, the *frequency max* map (Fig. 14) shows more than one value on the subject pixels, but demonstrates that the subject’s heart frequency was in an expected range for a sitting subject. Furthermore, a different frequency can be detected for the eyes. This is due to not only blinking, but also eye-movement which contributes to this signal. In addition, the subject suffers from an innate condition of nystagmus, where the eyes move rapidly horizontally, which could also have influenced the signal.

One notable observation is that some dynamic PPGI maps (e.g., *temporal variance*) allow the identification of objects in the background that should be static and, therefore, not visible in the maps. This manifestation could be due to the usage of inconsistent illumination probably caused by light sources which exhibit flicker. Furthermore, the left and right face halves show a different pattern in both PPGI cameras (e.g., *spectral flatness*, *spectral entropy*, *phasor phase* in Figs. 13 and 14, respectively).

Both *spectral flatness* and *spectral entropy* highlight the interesting regions by linking low values to pulsatile regions.

**Fig. 4:**
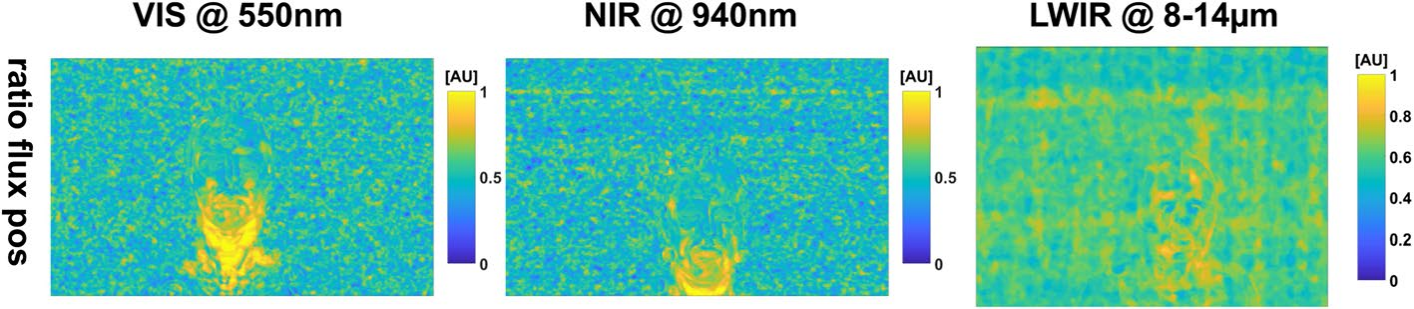
Enhancing small movement in the adult measurement w/o movement: a swallowing movement was made visible at the neck by the *spectral flux ratios* (*ratio flux pos*). The map for LWIR is distorted and, thus, it cannot be said whether the highlighting worked. However, the corresponding similarity map supports a highlighting (Fig. 16)

We can testify for PPGI that the pulsatile image segments contrast well against a noisy background: the background is characterized by a noisy pattern for all dynamic maps. It is also possible to see a horizontal line pattern (e.g., *spectral flux ratios* in Fig. 4) for the NIR camera which we attribute to the low lighting conditions and low saturation of the sensor. A similar pattern can also be seen for the IRT camera, though the origin is unknown.

*Enhanced dynamic movement* Even though the movement was aimed to be minimal, swallowing movement occurred during this particular time segment. The movement is visually enhanced by PPGI for the *spectral fluxes* and more so for the *ratios*, but not visible in IRT (Fig. 4). However, we think it is masked in IRT by background noise.

#### Similarity maps

**Fig. 5:**
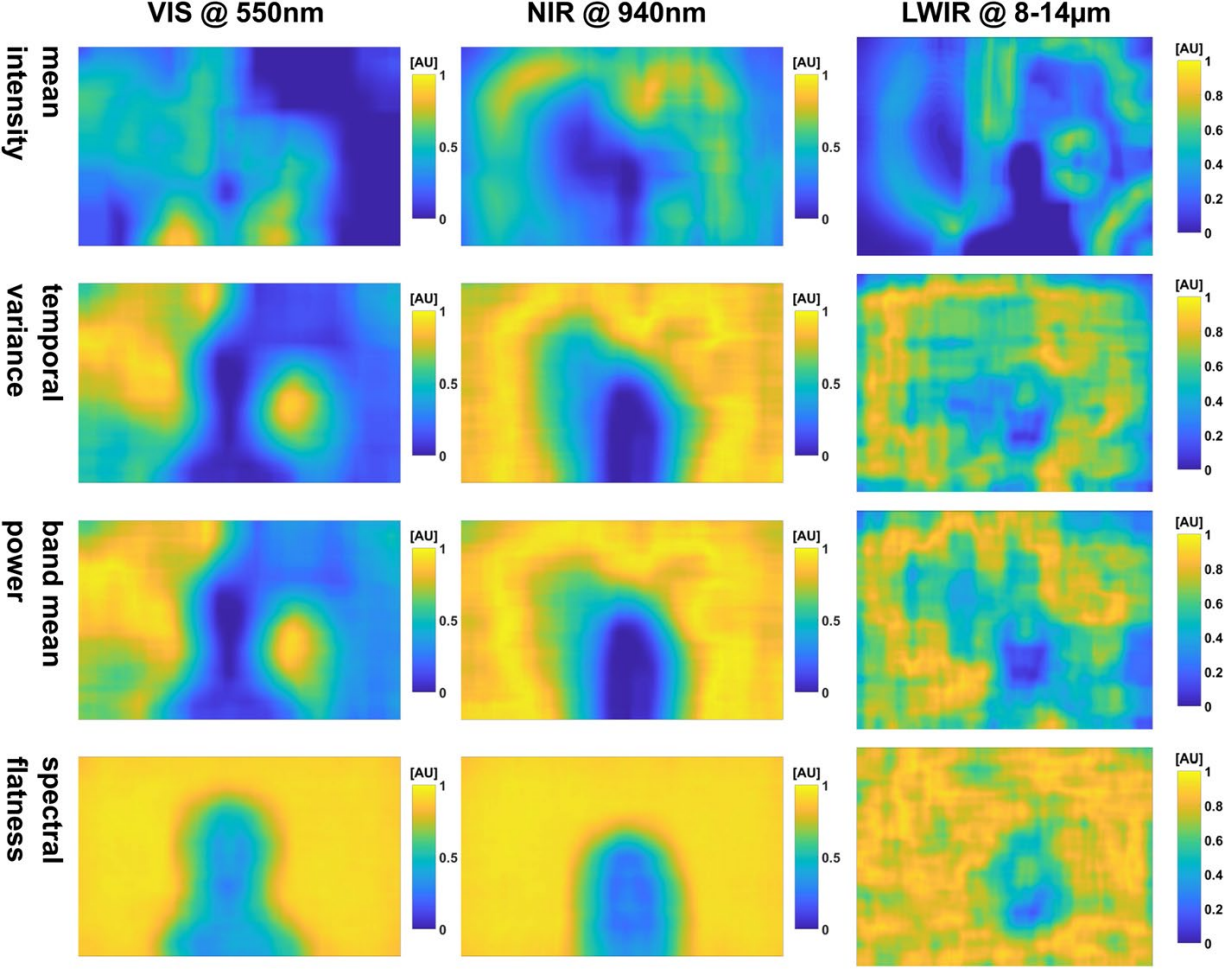
Similarity maps of the selection of feature maps of the adult measurement w/o movement: the *mean intensity* maps are not very uniform. The IRT map highlights the subjects silhouette, while the head is recognizable in the PPGI maps. The *temporal variance* and *band mean power* maps are less clear for all bands than the corresponding *spectral flatness* maps. The VIS maps especially suffer from an uneven distribution (left and right image halves have different histograms)

The maps are listed in Figs. 5,  15 and 16 in the appendix (Section C.1).

In general, the resulting maps of the histogram intersection approach (e.g., Fig. 5) expectedly show low similarity for subject pixels and higher values for the background. Note that the similarity maps depend on the selection of the ROI that is used for the histogram model. Hence, if a selected ROI does not model the background sufficiently, the similarity maps will show this difference. In addition, choosing an arbitrary region would result in completely different maps.

Regarding PPGI, the *mean intensity* map image regions are subjectively not as discernible as in IRT. As one can see, this approach is not suitable when the background is not noisy.

The dynamic maps of the *temporal variance* and *band power* allow one to discern both background and subject. However, in these maps, the background shows more variance compared to *spectral flatness*. Here, we can see clear advantages for similarity maps based on ‘non-decibel’ feature maps, such as the *spectral flatness*. The head is visible in IRT. However, the background is distorted, and discerning structures is harder than in PPGI.

#### Baby NICU measurement w/o movement

**Fig. 6:**
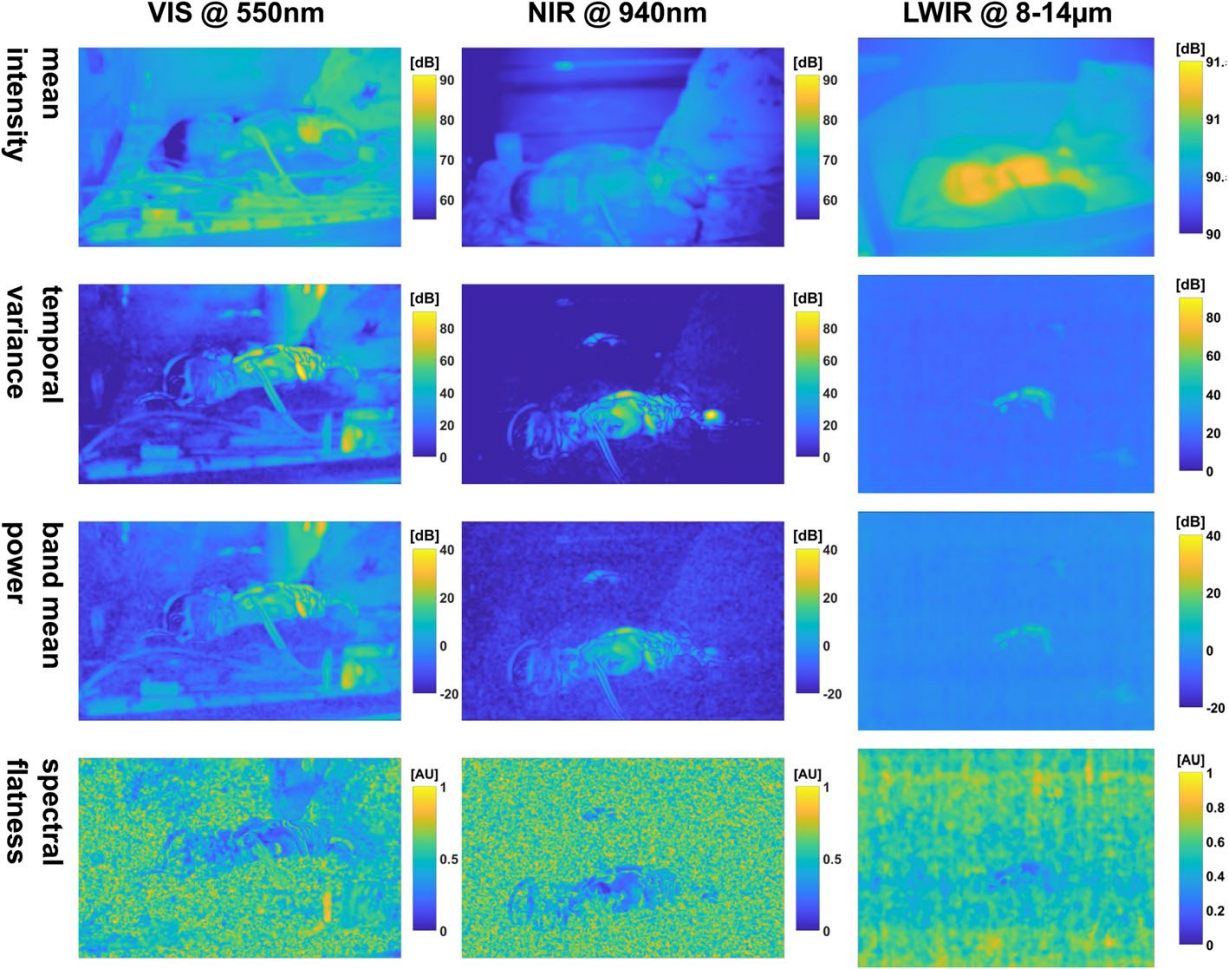
Selection of feature maps of the baby measurement w/o movement: *mean intensity* is used as a visual reference. All dynamic maps show a noisy background pattern. The VIS maps especially show additional image content, all of which is caused by cast shadows and reflections of the researcher behind the cameras of the measurement setup. A corresponding reflection in LWIR can be identified in the right bottom image corner of the dynamic maps. A reflection of the baby, at least in the PPGI maps, carries pulsatile signals and, thus, is visible above the abdomen

The maps for this video segment are given in Figs. 6, 21 and 22 in the appendix (Section C.1).

Many observations for the baby measurements are analogous to the adult measurement (e.g., signal power for the different cameras, background noise and faint visibility of the original background). One distinguishing fact about the background noise is the absence of the horizontal lines for the NIR camera, while the pattern in the IRT camera remains. The PPGI cameras again highlight more body parts (i.e., cover more body area), while the IRT maps only highlight the torso and belly, which are divided by a wire. A small round region below the torso can be observed which is an attached electrode.

We can identify multiple sources of pulsation by looking at the maps derived from the *power* (e.g., *band power*) in the heart band: The main source is the baby lying in the bed. Additionally, regarding the PPGI cameras, there are reflections of the baby on the plastic encasing the bed showing at least the torso. These reflections were also present in the IRT in the raw video.

When comparing the PPGI maps, we can also see that there is less power in the NIR maps compared to VIS, and that the baby is enhanced and only a noisy background is visible. By contrast, other sources of pulsation affect the VIS maps: There was movement behind the measurement setup during the recording. This becomes evident due to reflections at the front of the bed casing (right and left bottom) and next to the bed cover (right top), resulting in bigger and more connected structures compared to the background. These structures also have a similar high power in the same order as the pulsations of the baby (e.g., *band power*, *temporal variance*). Indeed, even parts of the bedcover are affected, probably due to the researcher’s shadow being cast.

The presence of the researcher can also be confirmed by the thermal camera showing a spot at the right bottom corner of the maps originating from the reflections of legs and shoes in the encasing. A second spot belonging to the researcher can also be seen above the belly of the baby (e.g., *band power*). We again ascribe the disturbance in PPGI to the illumination condition: while only one NIR source was used, not only the dedicated measurement light, but ambient light contributed to VIS. We can conclude that although the sources of disturbance are not in the field of view (FOV) of the cameras, undesirable image and map artifacts can occur.

Regarding fine details, the PPGI maps preserve, for example, the eyes. Bigger wires of skin-attached sensors are easy to spot and smaller wires generate a vessel-like pattern on the skin (due to movement). The *spectral fluxes* and *ratios* (Fig. 22) show changes for torso, belly and wires but the head is not visible, thus indicating only small changes. The smallest discernible object in the IRT maps is the electrode.

#### Similarity maps

**Fig. 7:**
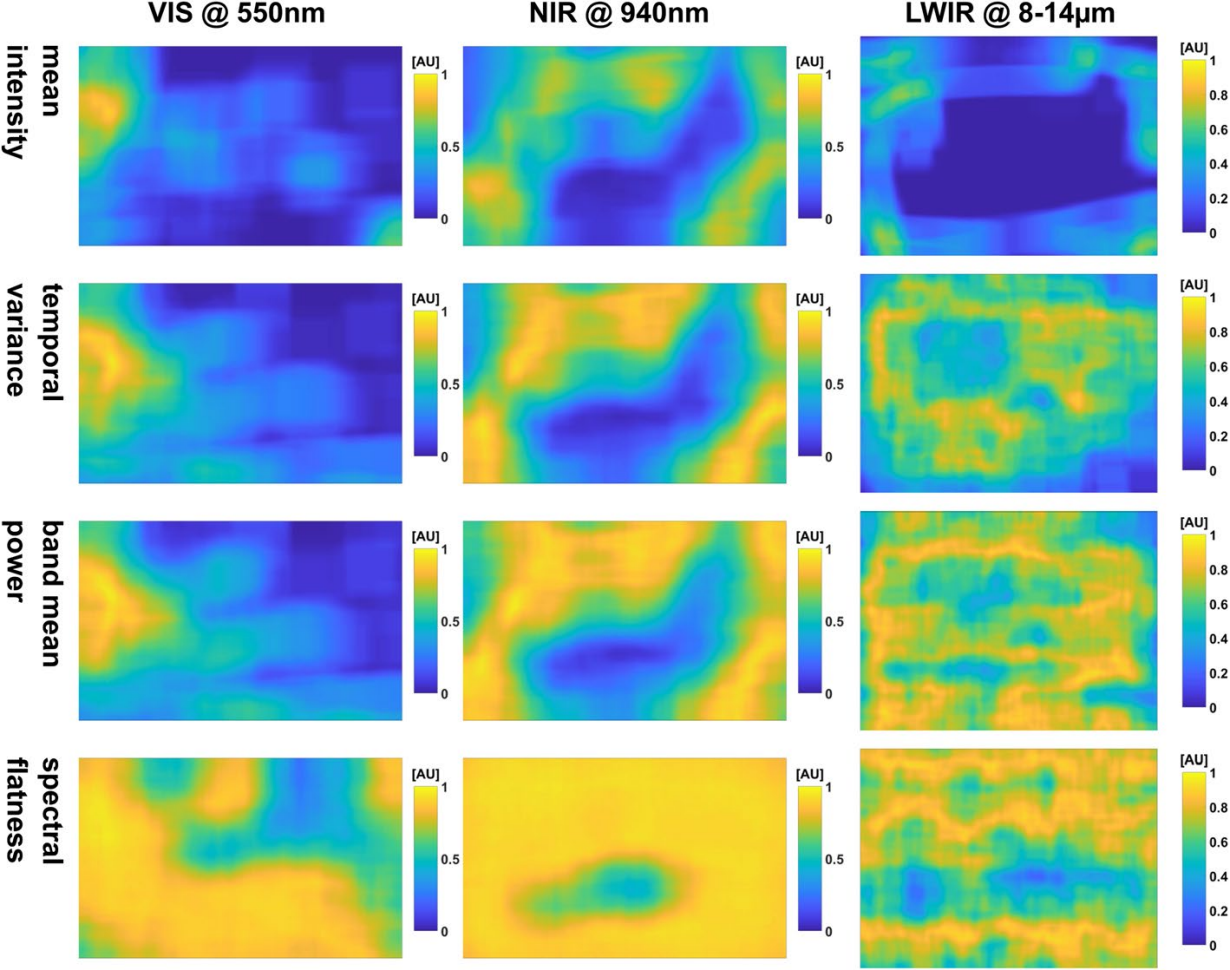
Similarity maps of the selection of feature maps of the baby measurement w/o movement: the *mean intensity* maps are not very uniform. It was harder here to select background regions for the histogram intersection approach. The VIS maps especially suffer from an inappropriate background model. Only *spectral flatness* highlights the location of the baby for this selection. The *spectral flatness* map in LWIR is also not visually clean. Nevertheless, low values for the similarity with the background were calculated for the pulsatile region. Hence, the pulsatile region is distinguishable

The observations made during the adult measurements are also true for the baby measurements (Figs. 7, 23 and 24 in Section C.1). However, due to the different settings, it was harder to define a big background region $ROI_\text {BG}$ (see Fig. 6). In addition to many reflective materials in the background, there was also the shadow cast by the researcher. Hence, ROIs were chosen retrospectively using the maps. The general results and especially those for VIS are not good due to the distortions. However, the position of the baby is recognizable at least for *spectral flatness* using NIR (see Fig. 7).

### RMS contrast

The results of the RMS contrast calculation for the adult and the baby measurements are given in Tables 1 and 2, respectively. We have highlighted the cases, where $ROI_\text {PULSE}$ has smaller values than $ROI_\text {BG}$ because we saw higher values for the majority of maps. The positions of the ROIs are highlighted in Section "Methods" (Figs. 11 and 12).

**Fig. 8:**
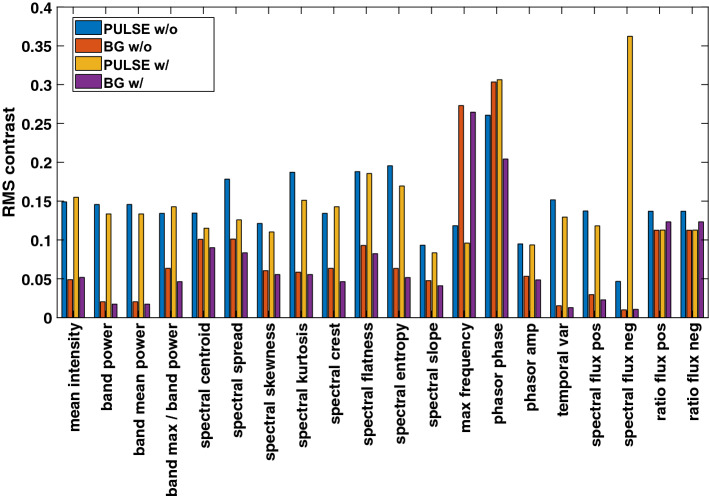
The RMS contrast of the adult measurement using NIR. The contrast for $ROI_\text {PULSE}$ is typically higher compared to $ROI_\text {BG}$. As expected, the results are inverted for *max frequency*. The complete set of feature definitions is given in Section B in the appendix. The location of the ROIs is given in Fig. 11

**Fig. 9:**
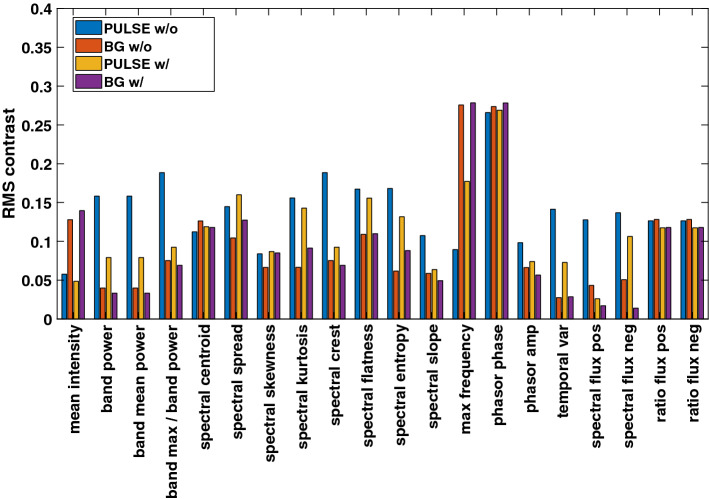
The RMS contrast of the baby measurements using NIR. The contrast for $ROI_\text {PULSE}$ is typically higher compared to $ROI_\text {BG}$. As expected, the results are inverted for *max frequency*. The location of the ROIs is given in Fig. 12

A graphical representation of the RMS results is presented exemplarily for the adult and the baby measurements using the NIR wavelength (Figs. 8 and 9): the RMS contrast is generally higher in pulsatile regions compared to non-pulsatile ones. This behavior is inverted for the *max frequency* maps.

In the case of movement in the adult and baby measurement, some maps show lower values in $ROI_\text {pulse}$ compared to $ROI_\text {BG}$ for VIS (e.g., *band power* according to Tables 1 and 2).

**Table 1:** The RMS contrast of the adult measurements: contrast per ROI without (w/o) and with (w/) movement

Map name	VIS				NIR				LWIR			
	w/o		w/		w/o		w/		w/o		w/	
	PULSE	BG	PULSE	BG	PULSE	BG	PULSE	BG	PULSE	BG	PULSE	BG
Mean intensity	0.1595	0.0370	0.1532	0.0380	0.1490	0.0489	0.1551	0.0518	0.2050	0.0531	0.1938	0.0536
Band power	0.1586	0.0240	*0.1823*	0.2276	0.1456	0.0204	0.1335	0.0173	0.2323	0.0284	0.1997	0.0131
Band mean power	0.1586	0.0240	*0.1823*	0.2276	0.1456	0.0204	0.1335	0.0173	0.2323	0.0284	0.1997	0.0131
Band max/band power	0.1141	0.0705	0.1388	0.0634	0.1342	0.0635	0.1428	0.0463	0.2398	0.0615	0.1877	0.0209
Spectral centroid	0.1686	0.1039	*0.1308*	0.1617	0.1346	0.1008	0.1152	0.0900	0.2315	0.0548	0.1863	0.0389
Spectral spread	0.1746	0.0961	0.1573	0.1060	0.1783	0.1012	0.1259	0.0834	0.2230	0.0598	0.1862	0.0454
Spectral skewness	0.1388	0.0777	0.1250	0.0914	0.1213	0.0604	0.1103	0.0554	0.2221	0.0437	0.1540	0.0241
Spectral kurtosis	0.2052	0.0734	0.1755	0.0983	0.1872	0.0585	0.1511	0.0555	0.2572	0.0295	0.1918	0.0308
Spectral crest	0.1141	0.0705	0.1388	0.0634	0.1342	0.0635	0.1428	0.0463	0.2398	0.0615	0.1877	0.0209
Spectral flatness	0.1993	0.1037	0.2027	0.1425	0.1881	0.0929	0.1856	0.0823	0.2361	0.0767	0.1975	0.0597
Spectral entropy	0.1858	0.0745	0.1606	0.0704	0.1955	0.0633	0.1695	0.0515	0.2749	0.0421	0.2025	0.0270
Spectral slope	0.1054	0.0488	*0.1097*	0.1485	0.0932	0.0476	0.0835	0.0410	0.1166	0.0585	0.0872	0.0471
Max frequency	*0.1702*	0.2685	*0.1100*	0.2768	*0.1183*	0.2731	*0.0960*	0.2645	*0.1944*	0.2974	*0.1283*	0.2154
Phasor phase	0.2838	0.2640	*0.3061*	0.3668	*0.2607*	0.3035	0.3064	0.2042	0.3175	0.0241	0.3108	0.0954
Phasor amp	0.1076	0.0572	*0.1030*	0.1154	0.0949	0.0532	0.0936	0.0485	0.1158	0.0234	0.1118	0.0356
Temporal var	0.1615	0.0172	*0.1843*	0.2361	0.1517	0.0153	0.1295	0.0129	0.2410	0.0253	0.2099	0.0113
Spectral flux pos	0.1539	0.0311	*0.1539*	0.2241	0.1373	0.0296	0.1181	0.0229	0.2107	0.0343	0.1791	0.0181
Spectral flux neg	0.0750	0.0098	0.3677	0.3521	0.0466	0.0100	0.3623	0.0106	0.2140	0.0508	0.2952	0.0042
Ratio flux pos	0.1482	0.1035	*0.1124*	0.2560	0.1370	0.1125	*0.1127*	0.1234	0.1409	0.1012	0.1696	0.0587
Ratio flux neg	0.1482	0.1035	*0.1124*	0.2560	0.1370	0.1125	*0.1127*	0.1234	0.1409	0.1012	0.1696	0.0587

The cases where the $ROI_\text {PULSE}$ has smaller values than $ROI_\text {BG}$ are highlighted. The complete set of feature map definitions is given in Section B in the appendix. The location of the ROIs is given in Fig. 11

**Table 2:** The RMS contrast of the baby measurements: contrast per ROI without (w/o) and with (w/) movement

Map name	VIS				NIR				LWIR			
	w/o		w/		w/o		w/		w/o		w/	
	PULSE	BG	PULSE	BG	PULSE	BG	PULSE	BG	PULSE	BG	PULSE	BG
Mean intensity	0.1247	0.0469	0.1149	0.0480	*0.0577*	0.1279	*0.0487*	0.1396	0.1226	0.0693	0.1012	0.0718
Band power	0.1621	0.0476	*0.0800*	0.0825	0.1583	0.0399	0.0791	0.0334	0.2582	0.0406	0.1429	0.0170
Band mean power	0.1621	0.0476	*0.0800*	0.0825	0.1583	0.0399	0.0791	0.0334	0.2582	0.0406	0.1429	0.0170
Band max/band power	0.1826	0.1102	0.1044	0.0705	0.1885	0.0754	0.0925	0.0692	0.2079	0.0723	0.1028	0.0555
Spectral centroid	*0.0654*	0.1616	0.1344	0.1196	*0.1122*	0.1263	0.1189	0.1180	*0.0554*	0.1107	0.1059	0.0530
Spectral spread	0.1412	0.1017	0.1319	0.1044	0.1447	0.1044	0.1600	0.1274	0.1884	0.0820	0.1883	0.0646
Spectral skewness	*0.0779*	0.0864	0.0833	0.0804	0.0840	0.0665	0.0869	0.0851	0.1683	0.0895	0.0711	0.0414
Spectral kurtosis	0.1456	0.0819	0.1202	0.1051	0.1559	0.0666	0.1428	0.0913	0.1889	0.0777	0.1304	0.0411
Spectral crest	0.1826	0.1102	0.1044	0.0705	0.1885	0.0754	0.0925	0.0692	0.2079	0.0723	0.1028	0.0555
Spectral flatness	0.1595	0.1130	0.1484	0.1158	0.1673	0.1090	0.1557	0.1099	0.1365	0.1030	0.1721	0.0799
Spectral entropy	0.1645	0.0727	0.1324	0.0954	0.1681	0.0618	0.1317	0.0881	0.1795	0.0657	0.1320	0.0456
Spectral slope	0.0959	0.0647	*0.0644*	0.0664	0.1074	0.0587	0.0637	0.0494	0.0986	0.0274	0.0746	0.0513
Max frequency	*0.0627*	0.3177	*0.1997*	0.2112	*0.0895*	0.2756	*0.1773*	0.2783	*0.0840*	0.1426	*0.1328*	0.1660
Phasor phase	0.2792	0.2632	*0.2553*	0.2909	*0.2658*	0.2736	*0.2689*	0.2783	0.3220	0.2951	*0.2433*	0.3364
Phasor amp	0.1003	0.0695	0.0737	0.0715	0.0982	0.0662	0.0740	0.0566	0.1236	0.0709	0.0883	0.0321
Temporal var	0.1657	0.0356	*0.0778*	0.0826	0.1415	0.0276	0.0729	0.0286	0.2721	0.0289	0.1355	0.0154
Spectral flux pos	0.0462	0.0138	*0.0257*	0.0296	0.1278	0.0432	0.0261	0.0170	0.2240	0.0567	0.1260	0.0206
Spectral flux neg	0.0421	0.0188	0.1441	0.0449	0.1370	0.0506	0.1063	0.0140	0.1759	0.0670	0.0441	0.0080
Ratio flux pos	*0.1274*	0.1329	*0.1110*	0.1591	*0.1265*	0.1282	*0.1174*	0.1179	*0.1014*	0.1128	0.0966	0.0791
Ratio flux neg	*0.1274*	0.1329	*0.1110*	0.1591	*0.1265*	0.1282	*0.1174*	0.1179	*0.1014*	0.1128	0.0966	0.0791

The cases where $ROI_\text {PULSE}$ has smaller values than $ROI_\text {BG}$ are highlighted. The location of the ROIs is given in Fig. 12

## Discussion

We looked at a selection of feature maps of two example scenarios in the previous section. Here, we will firstly discuss the key findings and the limitations of the approach presented.

Subsequently, we will suggest some recommendations for camera-based measurements on the basis of the measurements conducted and the results presented.

### Approach

The majority of feature maps allow us to visually discern the main source of pulsation (the subject silhouette (PPGI) or contour (IRT)) from the non-pulsatile background during small movements. The background is structured by a noisy pattern for all dynamic maps. We demonstrated that the histogram intersection approach helps us to explore the differences between spatial regions.

A smaller RMS contrast (standard deviation) compared to pulsatile regions could be observed for the majority of maps. In the case of the *max frequency* maps, the contrast is lower in the pulsatile regions. This observation supports the assumption that similar conditions exist within the pulsatile region.

We could identify some maps with lower contrast for the pulsatile region in VIS for the videos w/ movement. However, no further conclusions should be drawn as this is not consistent and we had only four samples (adult and baby measurement w/ and w/o movement). A direct comparison between the video segments w/o and w/ movement should particularly not be made since the value ranges are not identical in the maps (i.e., *min* and *max* pixel values are frame-dependent). However, the RMS contrast allowed us to detect differences between the two regions.

We could show that ‘non-decibel’ feature maps enable us to distinguish between pulsatile and background regions during small movements. Furthermore, we could observe, that the background is more similar and less structured than in ‘decibel’ maps. By contrast, during more intense movement, the ‘decibel’ maps allowed easier visual recognition of the subject’s position (see the appendix). However, this is to be expected when relatively long time segments (here, 10 s) are considered. In fact, more movement results in more changes and signal power. At the same time, the spectral distribution of the signal is different. Therefore, the ‘non-decibel’ maps (e.g., *spectral flatness*) are affected. In the future, this could be used to detect regions with movement artifacts.

What can be observed is that moving signal sources are spatially smeared, and, thus, affect relatively large areas. As an illustration, the feature maps of the adult measurement w/ movement (Fig. 17) visualize how a long time segment affects the maps: while hand and arm movements are not visible in the *mean intensity* map, all other maps show an effect. Movements that only contribute to some frames of a sequence are barely (or not) visible due to the mean filtering. Longer periods of movement, such as the ones observed at the baby (Fig. 12), blur the image regions so that fine details become unrecognizable. Adjusting the time window and the spatial parameters could mitigate the effect. However, these parameters need to be tuned per application.

Concerning the application of different wavelengths used for the modalities, we can see that the NIR in the clinical scenario, for example, is less affected by ambient light than VIS. Hence, NIR maps highlight the subject and not background sources. The IRT was not affected by light sources, at least in these measurements, but suffers from a noise pattern different from the one observed in the NIR. We recognized mainly contours in IRT, whereas in PPGI, silhouettes were visible, i.e., both modalities highlight different regions in the heart frequency band. Nevertheless, it is advantageous that the IRT maps were not disturbed by local illumination changes, which result from body movements. Consequently, the maps w/ movement highlight more subject pixels compared to the maps w/o movement (e.g., Figs. 21 and 25). Furthermore, the close surrounding of the subject is, at least visually, less influenced than what can be observed in PPGI.

We can conclude that the feature maps are useful to visualize certain aspects of the signal in the corresponding pixel region (similar to the Eulerian video magnification). Spotting regions of different frequencies (e.g., the eyes), is especially easy for a human using the maps. Furthermore, movement patterns, such as swallowing, can be made more perceivable (Fig. 4).

The similarity maps derived from feature maps also have useful functions. They allowed us to identify noise sources and suboptimal measurement conditions visually. The background needed to be visible in the scene for this. The maps displayed once more confirm the existence of a difference between pulsatile and non-pulsatile regions by highlighting the subjects. Moreover, the value range is between 0 and 1 for all maps. These maps could be exploited for segmentation using the same algorithm (regardless of the camera modality), in the future.

Consequently, we could show that there are advantages in not restricting the image to single ROIs at an early processing stage by exploiting the non-pulsatile background.

### Recommendations for camera-based measurements

The value of the maps is also determined by how they can improve camera-based sensing. We suggest the following recommendations based on the observations made in this work, especially for the acquisition of videos.

*Avoid reflective materials* As could be seen in the different maps, even reflections carry the pulsatile signal. On the positive side, this could be exploited to conduct indirect measurements or assess regions that are not directly visible (e.g., the body site not directed to the camera). On the negative side, reflective materials also reflect radiation from noise sources which may distort the signal in a particular ROI. Thus, when choosing a reference ROI to mitigate the effects of noise sources on a measurement ROI (e.g., [13]), calculating one of the maps described could help to determine good regions. If none are found, the setup can be changed (e.g., by adjusting the FOV).

*Avoid shadow casting* We tried to minimize the shadow cast in both experiments by using diffused light sources as the measurement light. However, the directionality of the sources was still visible and introduced unwanted effects. Hence, the common practice to record only the subject or disregard the background should be reconsidered to study the effects.

An evenly distributed and diffuse illumination is to be preferred to lessen the shadows. All light sources in the measurement environment should be taken into account, including ceiling lights, network activity lights, and computer or medical monitors.

*Choosing a signal quality index* Ratios of power are often used to assess the signal quality of a dynamic signal. However, we could see that it is harder to discern the pulsatile and non-pulsatile regions by using ‘decibel’ power maps exclusively. Indeed, other maps can be advantageous in these situations, for example, *spectral flatness* and *spectral entropy*.

*Camera parametrization* The extraction of dynamic information requires images free from motion blur. This means that long shutter times should be avoided whenever possible. Furthermore, automatic camera adjustments should be turned off. The sensor should be operated close to saturation to achieve good raw image material.

*Reference object* Depending on the application, it would be beneficial if a reference object was visible all the time. Such an object (e.g., grey card, color checkerboard, black body radiator) helps to acquire measurements in a consistent quality.

## Conclusions and outlook

We evaluated the generation of feature videos by transforming video segments into frames of new videos. The application intended is the sensing of vital signs using camera-based modalities such as PPGI and IRT. The feature videos can be used to assess which image regions of a video contain pulsatility. This is interesting, for example, because often only skin pixels are exploited for signal retrieval and non-skin pixels are discarded. However, we could show that useful signals are not only visible on the subject’s silhouette, but also in reflections or on nearby objects as well as on the contour of the subject.

As expected, a noisy image pattern, which defines non-pulsatile image regions, manifested in the background for both example measurements. The majority of maps showed a difference in the local RMS contrast in noisy vs. pulsatile regions. We successfully exploited the noisiness and could show that histogram intersection is a tool that could be used for further image segmentation. However, a suitable reference region had to be determined for building a model. Here, we had to manually select a region. This should be automated, and we suggest to either provide one or more suitable regions in the FOV of the camera during measurement (e.g., by using a grey card) or to focus on the development of a noise model of the background.

The maps show fine image details during small subject movements and more intense movements distort this information. However, we only generated maps from relatively long time segments (10 s) during which a lot of movement can occur. For shorter segments, the effect is expected to be weaker. Moreover, we suspect that the image acquisition parameters have a great impact on the maps. We think that each image should be free from motion blur, especially for the evaluation of dynamic phenomena, which means that the image integration time should be as short as the illumination conditions allow.

Regarding the application of different wavelengths to assess the activity in the heart frequency range, we could show that despite the lower sensitivity of silicon in the NIR range, these maps can be less disturbed than visual ones because fewer NIR sources add to ambient light for the scenarios given.

The PPGI generally highlights the silhouette, while contours are emphasized for IRT. We particularly found high power at the head contour (adult) and torso (baby). This is in accordance with previous findings, at least for adults, which suggest that head movement can be a result of ballistocardiographic blood pulsations [11, 34]. However, these high-contrast image regions at the head contour might not be suitable to extract the pulse rate [21] but to locate the head.

We showed the adverse effects of shadow being cast and reflective materials which should be considered when deploying camera-based sensing technologies or need to be considered in algorithmic development. Algorithms that rely on reference regions for artifact removal could especially benefit from choosing regions accordingly.

In this work, we could not determine a ‘best’ map. However, some maps have properties that are desirable, such as a well-defined value range. It should be noted that the temporal filtering and frequency transforms are computationally expensive and account for the biggest share in computational time (excluding the very expensive spatial pooling operation) and scale with the number of signals, i.e., with the spatial resolution of the maps. Furthermore, there is an overhead for calculating the PS versus the FFT-spectrum. The actual feature computation is performed comparatively fast.

While most maps presented compress the information of a single time segment, the spectral flux ratios combine two subsequent segments. Hence, more dynamic information is compressed. As a secondary result, the flux ratios proved to be valuable tools for the visual detection of movement activity.

Future work could direct research on which maps could be exploited best and if certain regions correspond to those that, for example, deep learning approaches would use for vital signs retrieval.

However, calculation of the maps is still very time consuming with the current processing capabilities and this restricts the spatial output resolution if real-time processing is required. We could make new observations and gained valuable insights for the deployment of camera-based sensing in realistic scenarios by computing feature maps at a high resolution. The approach described just relies on the temporal dynamic characteristic of the signal and, thus, should be exploitable for various imaging applications.

## Methods

In this section, the processing chain is described in more detail. Furthermore, the visualization of feature maps and the postprocessing steps for evaluation are explained. Finally, the videos used for evaluation are presented.

### Processing chain

A sketch of the generation of feature videos is provided in Fig. 1: each ROI of a video segment of fixed length contains a time series. Features of the time series or from its spectral representation (spectrum) can be mapped to the corresponding ROI position and, thereby, create a new image. Subsequent processing of video segments results in a feature video.

The processing chain can be decomposed into three main stages mandatory for generating feature videos and two other stages dealing with pre- and postprocessing (Fig. 2):

- Firstly, pixels are spatially pooled and the resulting images are subsampled.
- Each pixel then represents a ROI for which a temporal filtered signal is created.
- Depending on the feature, the signal is statistically evaluated (temporal feature) or transformed into the frequency domain beforehand.

An optional preprocessing replaces dropped frames by a simple linear interpolation using neighboring frames. The postprocessing stage describes how the maps are evaluated in this work.

The parametrization used for each stage is given in Table 3.

The processing chain was implemented in Matlab 2017b.

**Table 3:** Processing chain parameters used for the chain given in Fig. 2

*Preprocessing*			
Method linear interpolation			
$N_{\text {samples}}$ all			

*ROI generation*			
	*PPGI*		*IRT*
*Spatial pooling*			
Filter			
Type		Gaussian	
$\sigma _\text {gauss}$	10		5
$N_\text {kernel}$	41		33
*Decimation*			
$d_\text {x}$	5		1
$d_\text {y}$	5		1
$b_\text {center}$		True	

*Temporal filtering*			
$T_\text {seg}$	10		
$N_\text {seg}$	250		
Filter			
Type	IIR		
Design	Butterworth		
$N_\text {order,design}$	6		
$b_\text {zero-phase}$	True		
$f_{\frac{1}{2}\text {,low,baby}}$	0.80 Hz		
$f_{\frac{1}{2}\text {,high,adult}}$	5 Hz		
$f_{\frac{1}{2}\text {,low,baby}}$	1.30 Hz		
$f_{\frac{1}{2}\text {,high,adult}}$	5 Hz		

*Feature generation*			
Window			
Type	Hann		
$N_\text {win}$	250		
$N_\text {overlap}$	249		
$N_\text {FFT}$	1024		
$b_\text {zero-pad}$	True		
$f_{\text {FFT low, adult}}$	0.85 Hz ($\approx$50bpm)		
$f_{\text {FFT high, adult}}$	3.98 Hz ($\approx$240bpm)		
$N_{\text {band, adult}}$	129		
$f_{\text {FFT low, baby}}$	1.51 Hz ($\approx$90bpm)		
$f_{\text {FFT high, baby}}$	3.66 Hz ($\approx$220bpm)		
$N_{\text {band, baby}}$	89		

*Postprocessing*			
*Histogram intersectio*n			
$N_{\text {scale}}$	256		
$N_{\text {bins}}$	256		
$N_{\text {kernel,hist}}$	31		
Padding	Symmetric		

#### ROI generation

Each image is spatially filtered by a Gaussian filter. Thus, the resulting image is a blurred version of the input image and the resulting pixels represent spatially weighted averages of their respective surroundings. A subsequent subsampling (decimation) reduces the image dimensions. Consequently, we still retain a regular grid (here, Cartesian) which can be evaluated. The Gaussian weighting ensures that the central pixel contributes more highly to the output pixel value and in contrast to a uniform mean weighting scheme, the resulting images are not blocky. Subsampling can be applied to reduce the computational load in the subsequent stages at the expense of a loss in spatial resolution.

Choosing the right parameters for spatial filtering is application-dependent. However, a certain number of pixels generally need to be combined to form a time series and stronger blurring reduces the effects of small movements at the expense of spatial resolution. Similarly, a lower subsampling will result in higher resolution but, simultaneously, more signals need to be processed.

#### Temporal filtering

In the second stage, each pixel time series is evaluated by a sliding window approach. The respective temporal mean value is subtracted in each time window (segment). Afterwards, a temporal bandpass filter is applied to limit the signal to a pre-defined frequency band of interest. Here, this is the range of anticipated heart frequencies. In this paper, we use forward–backward filtering with an infinite impulse response (IIR) filter. The signals are then windowed by a windowing function (Hann window).

Because a high number of signals need to be filtered, the filter length should be kept short. Thus, IIR filters are a suitable choice.

#### Feature generation

A frame of a feature video is created by calculating a feature on each pixel series and simply mapping it on the ROI position on the image grid. In this work, the temporal signal variance within a time segment $s_\text {seg}[n]$ is the only dynamic temporal feature computed. All other features are spectral features calculated from the frequency domain using one or two time segments. The windowed segments are zero-padded to a length equal to a natural power of two ($N_\text {FFT}=1024$). To clarify, the previous processing steps are used to prepare for calculating the single-sided discrete Fourier transform (DFT) and the power spectrum (PS) ($\equiv 2\cdot |DFT|^2$ scaled by the inverse of the squared sum of the window values [35]) of the time series or its segments $s_\text {seg}[n]$. To be more precise, we are calculating the short-time Fourier transforms (STFTs) of the time series for a whole video, where each time segment has a corresponding DFT and PS segment. The segment is padded accordingly to apply the fast Fourier transform (FFT) algorithm, instead of the slower DFT.

Regarding the spectral features, a narrow band is defined by a lower and upper frequency bin $b_\text {low}$ and $b_\text {high}$ corresponding to the frequency range selected $f_\text {low,subject}$ and $f_\text {high,subject}$. Thus, the number $N_\text {band}$ of discrete spectral bins ($[b_\text {low},b_\text {high}]$) depends on the frequency range (Table 3 and Fig. 1).

#### Feature description

For the sake of readability, the feature definitions can be found at the end of the paper in the appendix (Section B).

We generated a total of 20 different maps: nineteen feature maps and one map as a visual reference. The *mean intensity* of the video sequence that was subtracted during the temporal filtering (Fig. 2) was used as a visual reference.

The general idea is that when a pulsatile signal is present, the features are different from the case when such a signal is absent.

In the case of the spectral features, each spectrum can be viewed as a (non-normalized) distribution. The spectral features are tools to describe the shape, center, spread, and other characteristics of the distributions and, therefore, should allow the differentiation of pulsatile from noise-like signals. Furthermore, if there is temporal variance in the filtered time series, it can indicate the presence of pulsatility.

In particular, spatial regions corresponding to the same signal source should be similar (values of the features are in similar ranges). We will explore this further below visually and by a measure of similarity.

### Visualization

Not all maps are visually appealing (e.g., these are too dark to allow the discernment of details) without an appropriate scaling. For these maps, a logarithmic conversion was applied beforehand as given below:

1 $$\begin{aligned} V\left( I\right) =U\cdot \text {log}_{10}\left( |I|+\varepsilon \right) [\text {dB}], \end{aligned}$$

where $\varepsilon$ represents a small number to handle the case where a map pixel is zero, *I* is an image/map and $U\in \{10,20\}$ is a factor depending on the map for the correct conversion to decibel.

Afterwards, a separate windowing has been applied for each image to display the various maps. In each case, this was a linear gray-scale transform that maps the pixel values to limits selected manually. Values outside the range were clipped to the lowest and highest values of the windowing range to map all pixels. The limits were set manually to allow a visual comparison.

### Postprocessing

Subsequent segmentation of the maps is necessary to identify sources and corresponding spatial image locations. However, this would be a topic on its own. For now, we just wanted to find out whether the background distinguishes from pulsatile image regions.

#### Similarity measurement

We compared histogram intersections similar to [36] to measure the similarity. We defined a normalized model histogram $H_M$ and compared it to the normalized histogram of an image patch $H_P$. Hence, the intersection $\eta (H_M,H_P)$ is:

2 $$\begin{aligned} \eta (H_M,H_P) = \sum _{j=1}^{N_\text {bins}}\text {min}(H_{M,j}H_{P,j}). \end{aligned}$$

**Fig. 10:**
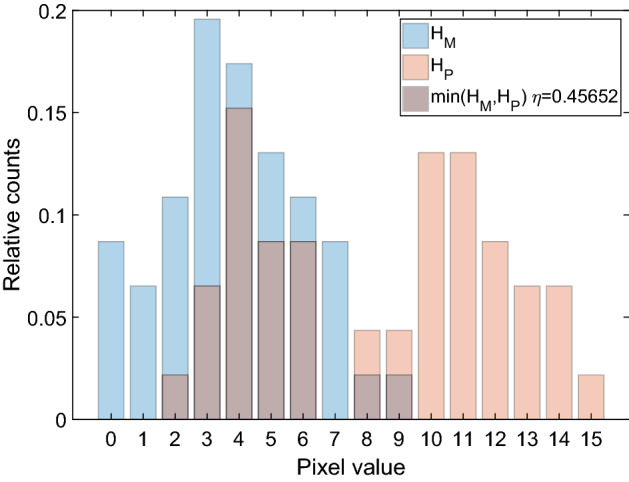
Histogram intersection as a measure of similarity. We created two simplified histograms ($H_M$ and $H_P$) with 16 ($N_\text {bins}=16$) different pixel values to visualize (2) and the calculation of $\eta$

Values of $\eta$ range between [0, 1]. To demonstrate the calculation of $\eta$, Fig. 10 is provided.

We have constructed ‘similarity maps’ by calculating $\eta$ for each image patch of a map. Consequently, the maps were discretized to $N_\text {scale}=N_\text {bins}=256$ levels. We tried to pick a reasonably big and, thus, representative background region to build the model histograms for each camera. Here, this region was the same for all maps of a camera. A histogram was then computed for each pixel from an image patch, a $31 \times 31\hbox { pix}$ block ($N_\text {kernel,hist}=31$) with the pixel in the center. Symmetric padding was used to handle boundary pixels. We implemented a naive and slow approach for the calculation of the histogram intersections. However, a much faster implementation could be possible by calculating so-called ‘integral histograms’ [37].

### Evaluation

As stated above, the value ranges of the individual maps differ; for example, some are bound while others are not. Thus, the values were scaled to $N_\text {scale}=256$ levels to make the maps comparable. However, the scaling was normalized to the range [0, 1] using all the pixels of a map.

We chose to compare manually selected ROIs by calculating the spatial standard deviation (SD) to find out whether there is not only a visual difference between pulsatile and non-pulsatile image regions.

***RMS contrast*** The SD is also known as the RMS contrast $C_\text {RMS}$ [38]:

3 $$\begin{aligned} C_\text {RMS}=\sqrt{\frac{1}{N_\text {pix}-1}\sum _{i=1}^{N_\text {pix}} \left( A_{i}-\mu _\text {pix}\right) ^2}, \end{aligned}$$

where $\mu _\text {pix}$ is the mean of all (${N_\text {pix}}$) pixels in the ROI and *A* is the vector containing all pixels of said ROI.

The same representative ROIs have been processed for each map: One very large region represents the background pixels ($ROI_\text {BG}$), the other region has been chosen to carry the pulsatility of a signal ($ROI_\text {PULSE}$). The $ROI_\text {BG}$ was also used to calculate $H_M$ for all maps.

We calculated the RMS contrast for images of the video sequences described below. Two time segments were selected per video: One without (w/o) and one with (w/) movement.

#### Video sequences

We used videos of two measurements serving as illustrative examples for the evaluation: 1) An adult in a controlled lab and 2) a baby in a clinical scenario. The cameras used for the measurements and the resulting feature map resolutions are provided in Table 4, while the measurements are described below.

An overview of the camera setup is given in [39]. Due to the setup, the cameras have a different FOV and parallax is present between the cameras.

**Table 4:** Cameras used for the recordings

Modality	Optical band	Type	Fps $f_s$	Resolution	Mode	ADC	Lens	Optical filters	Model	Map resolution
			[Hz]	$[\hbox {pix}] \times [\hbox {pix}]$		[bit]	[mm]			$[\hbox {pix}] \times [\hbox {pix}]$
PPGI	VIS	Mono	25	$1920 \times 1200$	7	12	12.5	Green narrow bandpass; 550 nm	Grasshopper 3 GS3-U3-23S6M-C	$376 \times 232$
	NIR	Mono	25	$1920 \times 1200$	7	12	12.5	NIR narrow bandpass; 940 nm	Grasshopper 3 GS3-U3-23S6M-C	$376 \times 232$
IRT	LWIR	LWIR	25	$640 \times 480$	NA	16	10	-	Gobi-640-GigE	$608 \times 448$

**Fig. 11:**
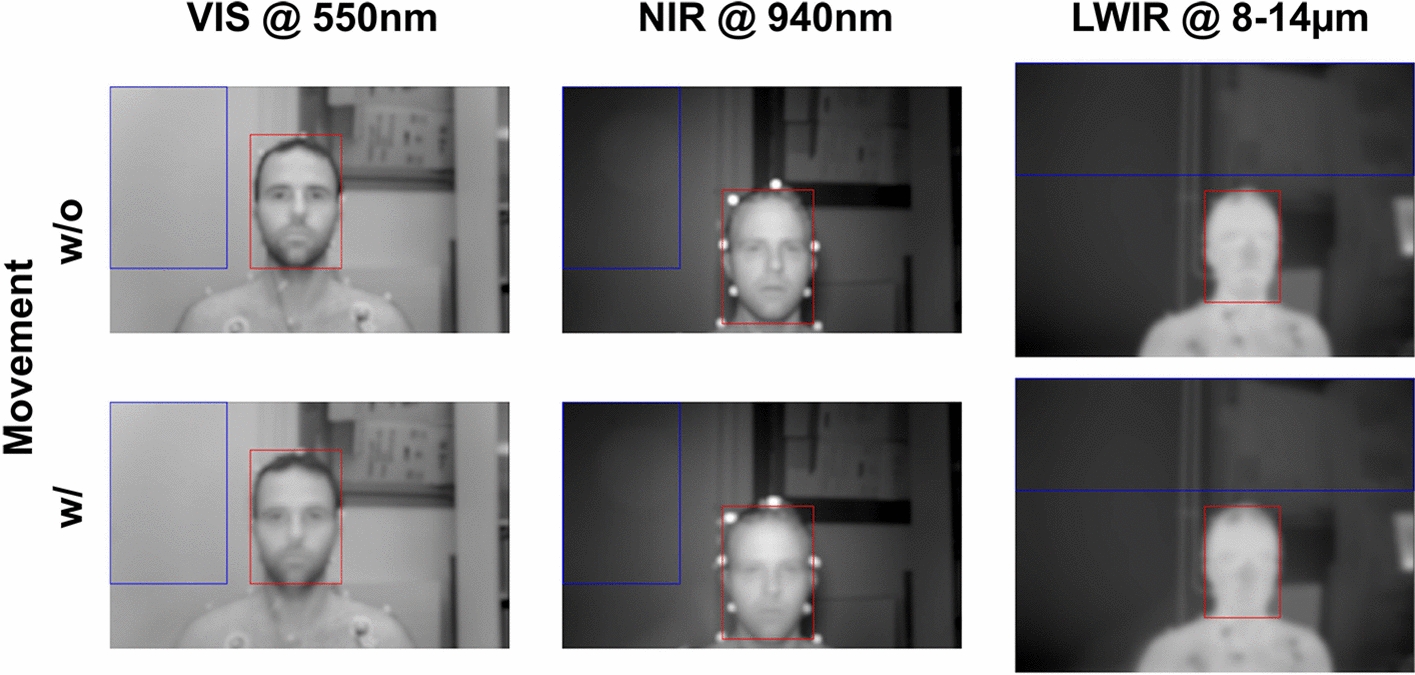
Adult face video *w/o* (top) and *w/* movement (bottom): *mean intensity* mapping of 10-s segments. The round optical markers are doubled due to movement. Furthermore, motion blur reduces the detail in the facial region. The movement of the arm is not visible due to the temporal low-pass effect (bottom). The ROIs marked for evaluation were manually chosen and correspond to the background ($ROI_\text {BG}$; blue) and to a region that was expected to show pulsatility ($ROI_\text {PULSE}$; red)

*Adult* In this scenario, an adult subject with an unclothed upper body had been sitting on a chair and was asked to sit still to conduct a video recording (Fig. 11, top). As part of the measurement, the subject took a small spoon of chili sauce (Fig. 11, bottom). Consequently, movement in this scenario is expected to originate from breathing movements, eye movements, swallowing and the changing of facial expressions. Moreover, the movement sequence also contains hand to face movement when consuming the sauce.

Such a measurement is comparable to facial measurements of the pulse rate using webcams. However, more skin pixels are present due to the unclothed torso. Furthermore, spherical optical markers, which are highly reflective in the NIR, were attached to the head and upper torso. The markers were only used here as visual cues.

**Fig. 12:**
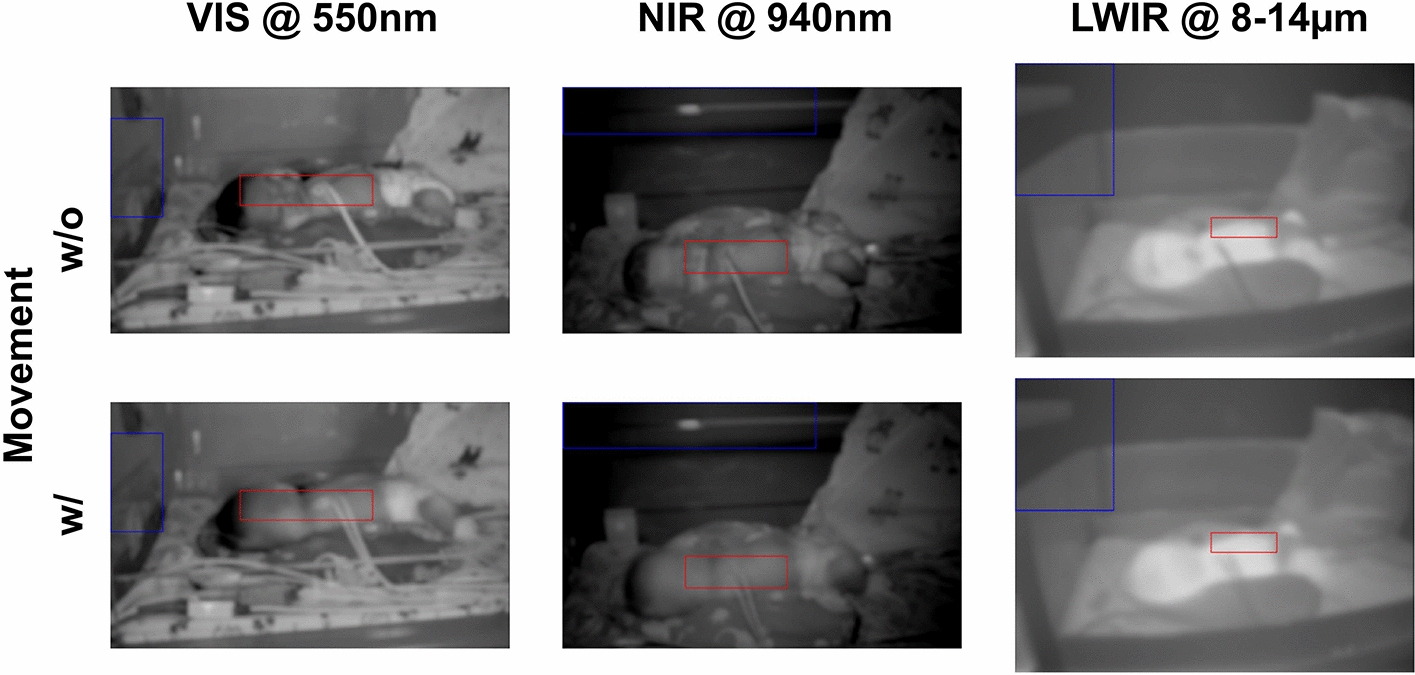
Video of a baby in a thermal bed *w/o* (top) and *w/* movement (bottom): *mean intensity* mapping of 10-s segments. In the segment w/ movement, motion blur reduces all details in the face. The ROIs marked for evaluation were manually chosen and correspond to the background ($ROI_\text {BG}$; blue) and to a region that was expected to show pulsatility ($ROI_\text {PULSE}$; red). The regions selected are different for the three cameras to account for the different fields of view and disturbances detected in the video streams (i.e., shadows cast, reflections)

**Fig. 13:**
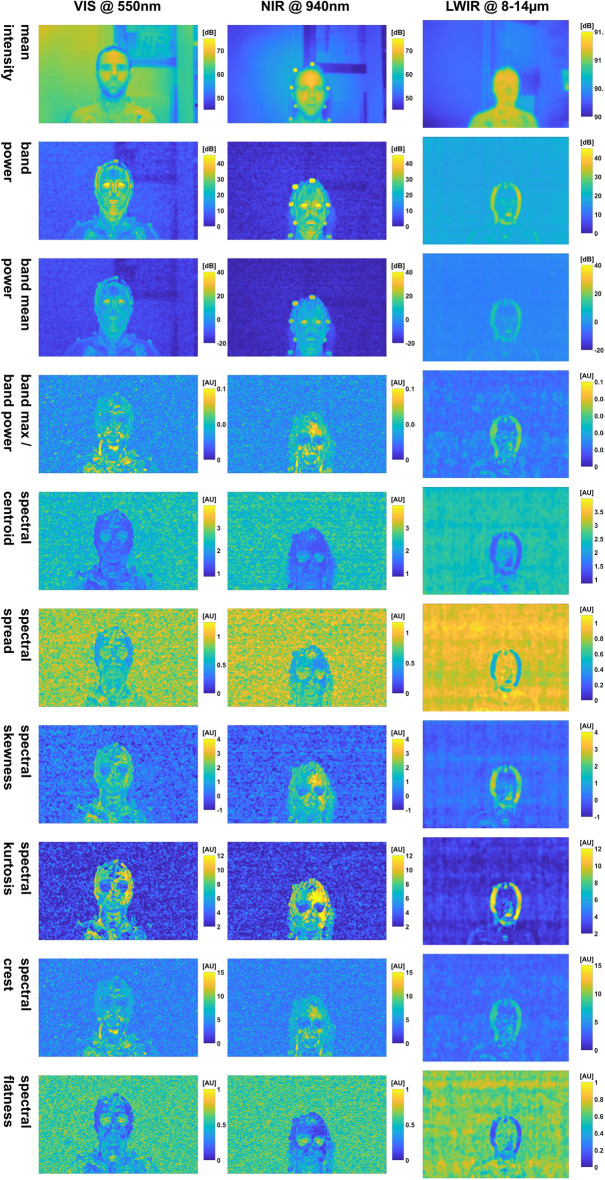
Feature maps of adult measurement w/o movement: *mean intensity* to *spectral flatness*

**Fig. 14:**
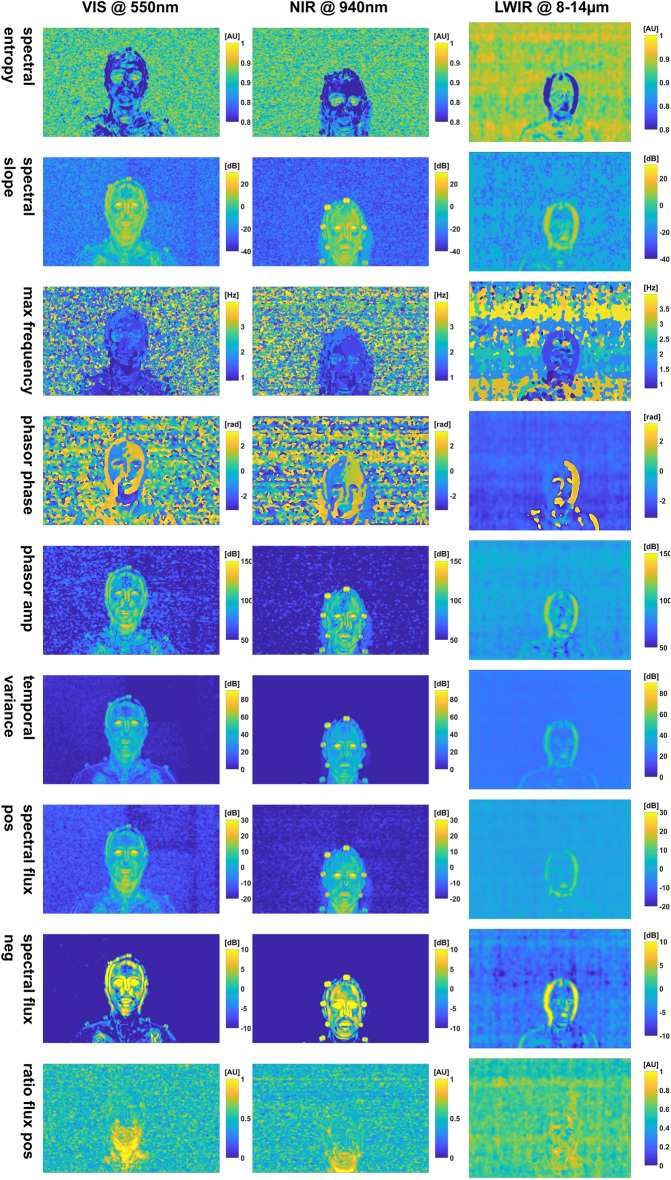
Feature maps of adult measurement w/o movement: *spectral entropy* to *flux ratios*

**Fig. 15:**
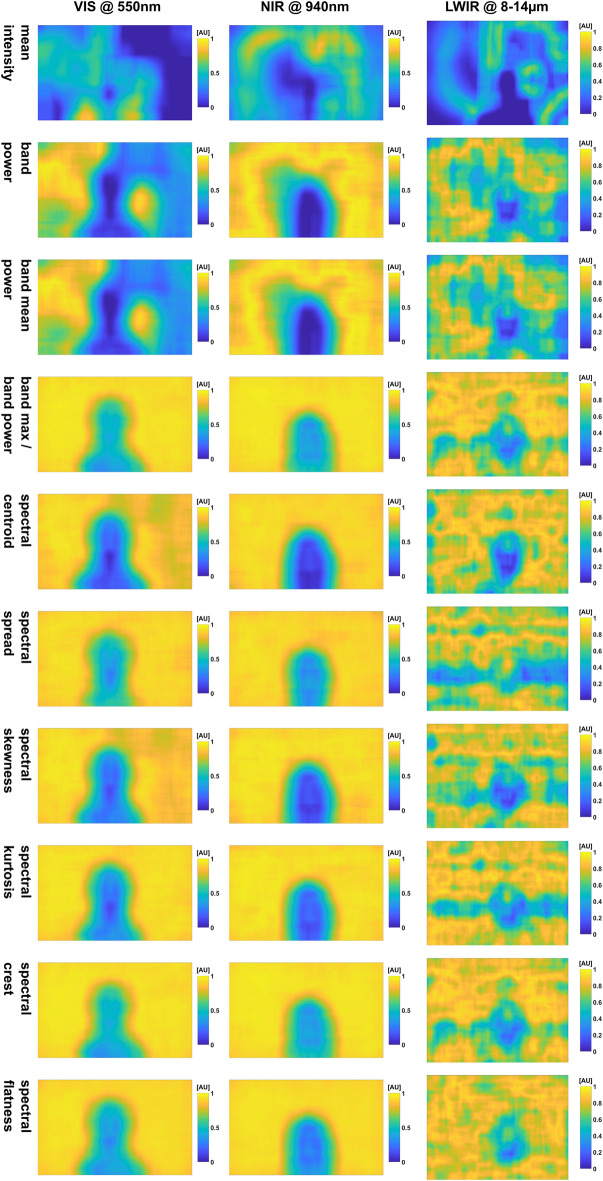
Similarity maps of adult measurement w/o movement: *mean intensity* to *spectral flatness*

**Fig. 16:**
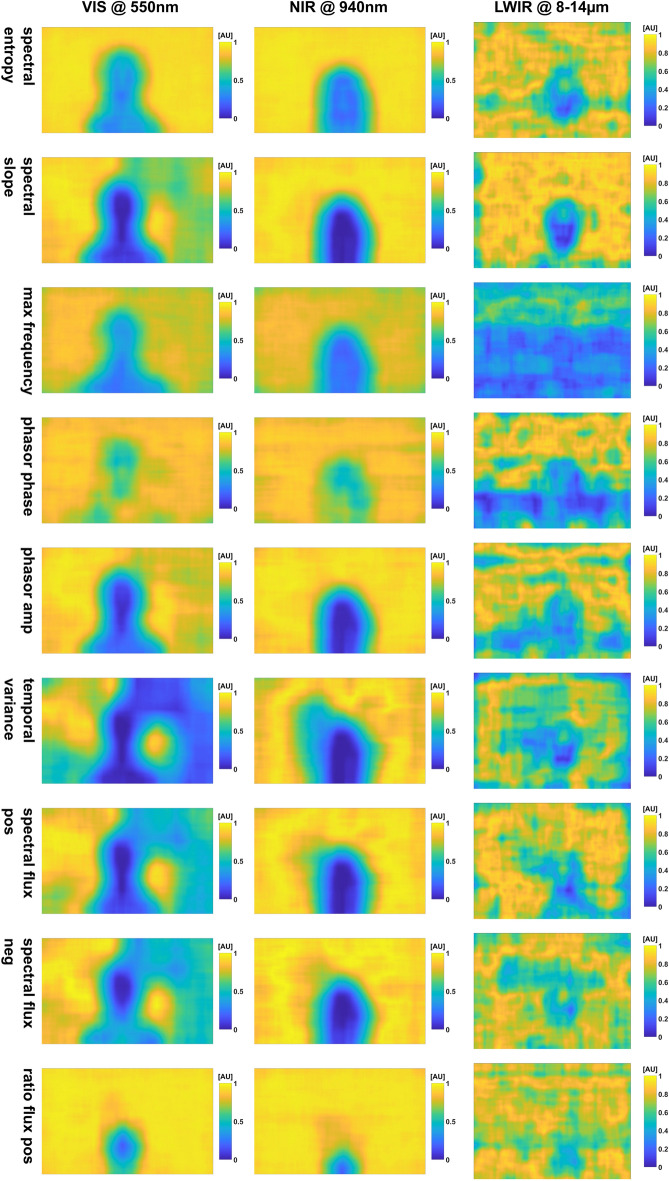
Similarity maps of adult measurement w/o movement: *spectral entropy* to *flux ratios*

**Fig. 17:**
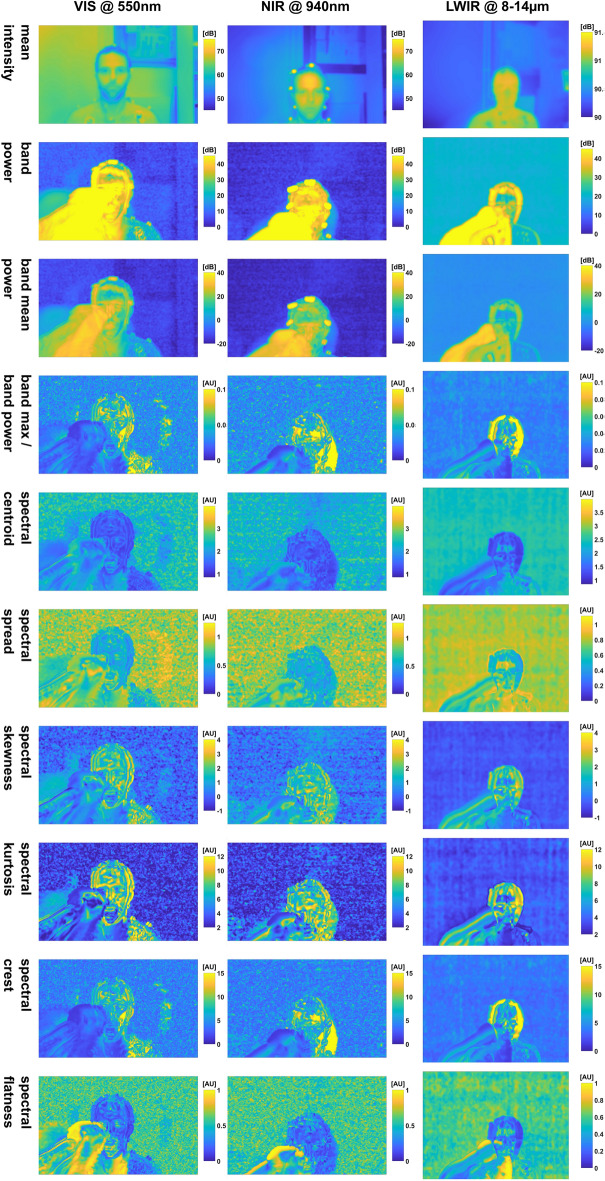
Feature maps of adult measurement w/ movement: *mean intensity* to *spectral flatness*

**Fig. 21:**
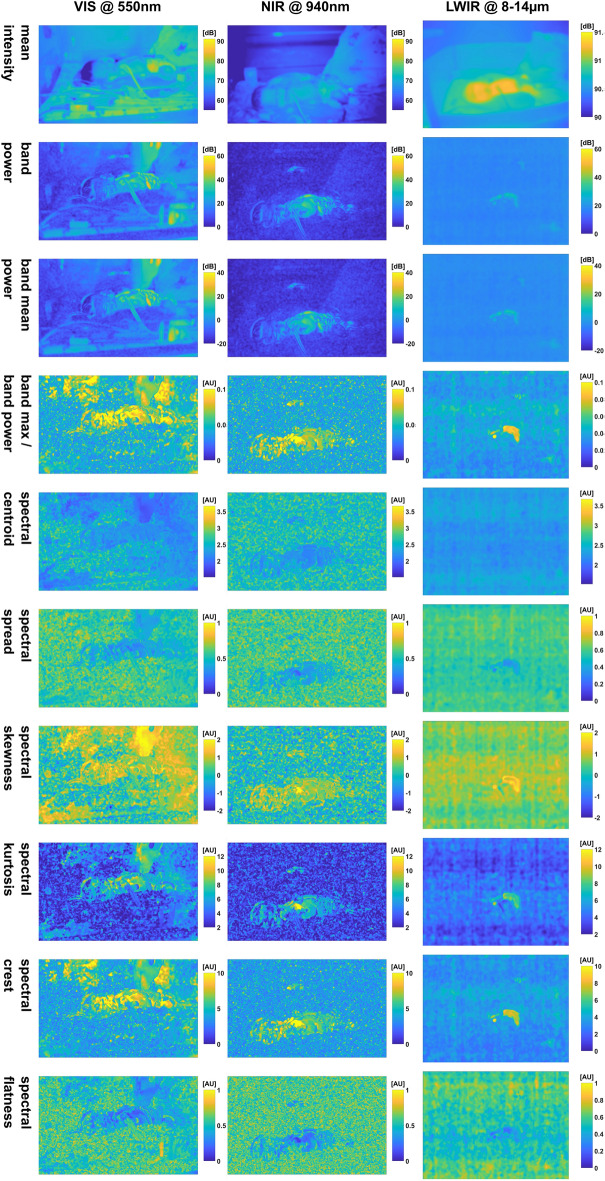
Feature maps of baby measurement w/o movement: *mean intensity* to *spectral flatness*

**Fig. 22:**
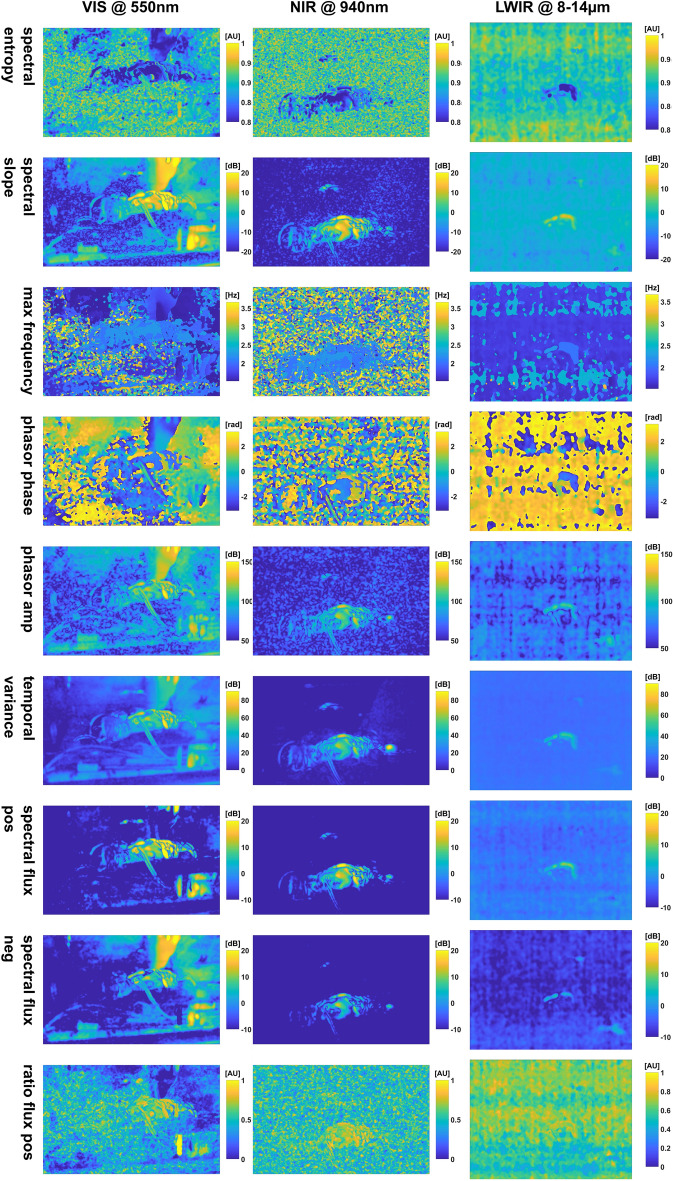
Feature maps of baby measurement w/o movement: *spectral entropy* to *flux ratios*

**Fig. 23:**
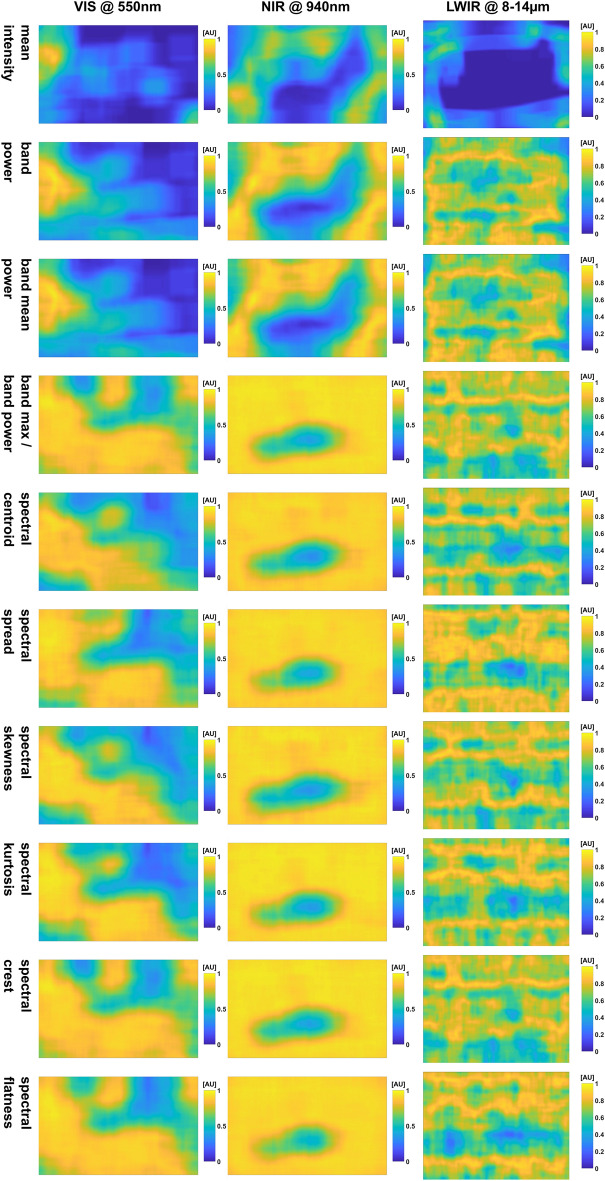
Similarity maps of baby measurement w/o movement: *mean intensity* to *spectral flatness*

**Fig. 24:**
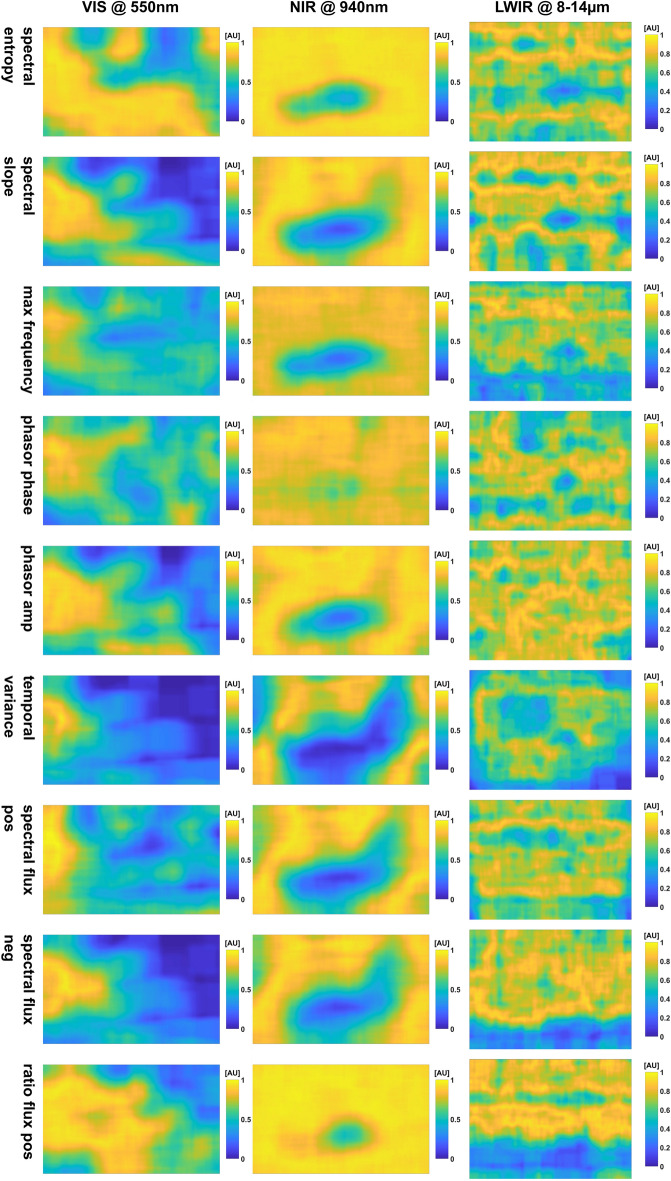
Similarity maps of baby measurement w/o movement: *spectral entropy* to *flux ratios*

**Fig. 25:**
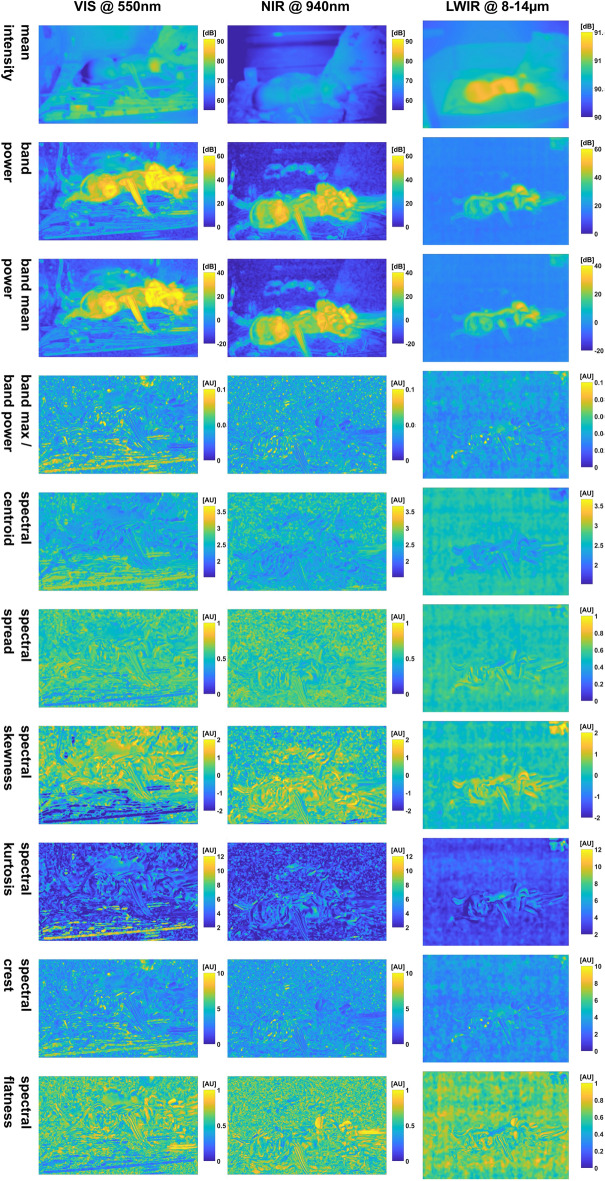
Feature maps of baby measurement w/ movement: *mean intensity* to *spectral flatness*

*Baby* Whereas the first scenario was very controlled, the recordings of a baby in the clinic (Fig. 12, top) were more affected by movement (Fig. 12, bottom): the baby was moving randomly and, thus, changed the illumination conditions at various image regions. Furthermore, the illumination was influenced by people not directly visible in the scene but who cast shadows and were also visible in mirror-like reflections of the subject’s bed. To be more precise, people were moving behind the measurement setup.

## Figures and Tables

**Fig. 18:**
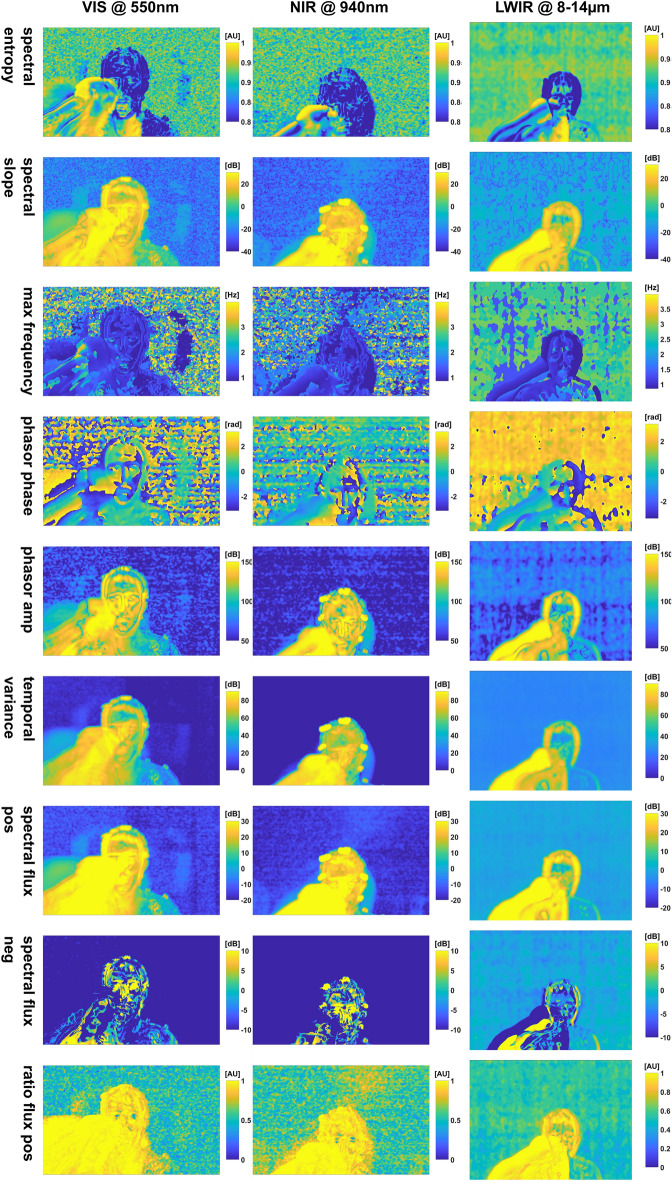
Feature maps of adult measurement w/ movement: *spectral entropy* to *flux ratios*

**Fig. 19:**
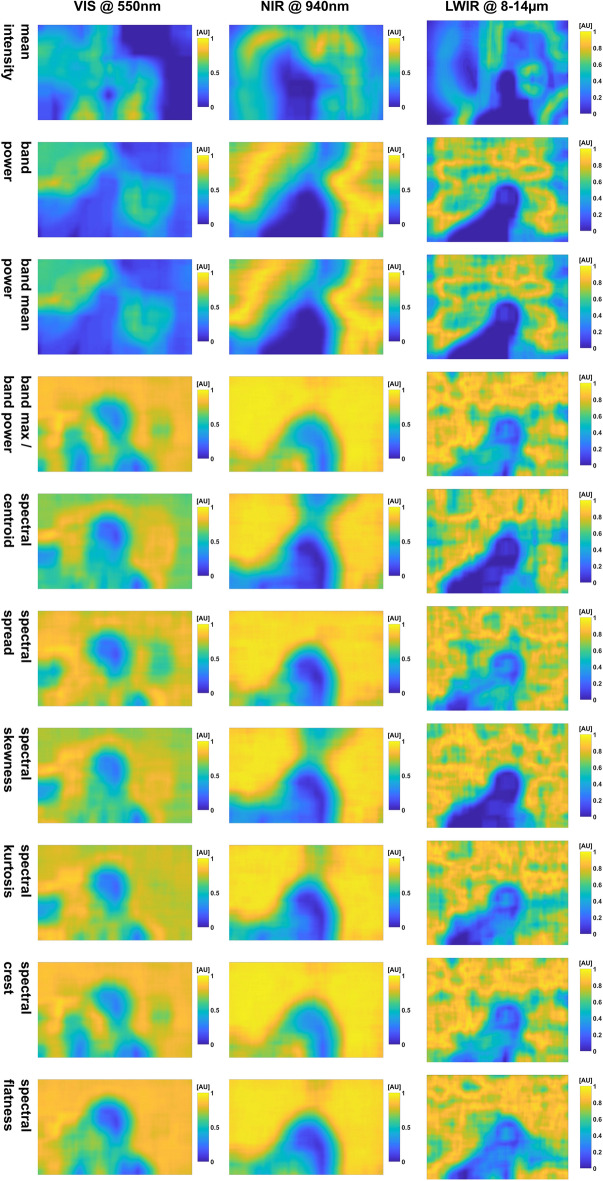
Similarity maps of adult measurement w/ movement: *mean intensity* to *spectral flatness*

**Fig. 20:**
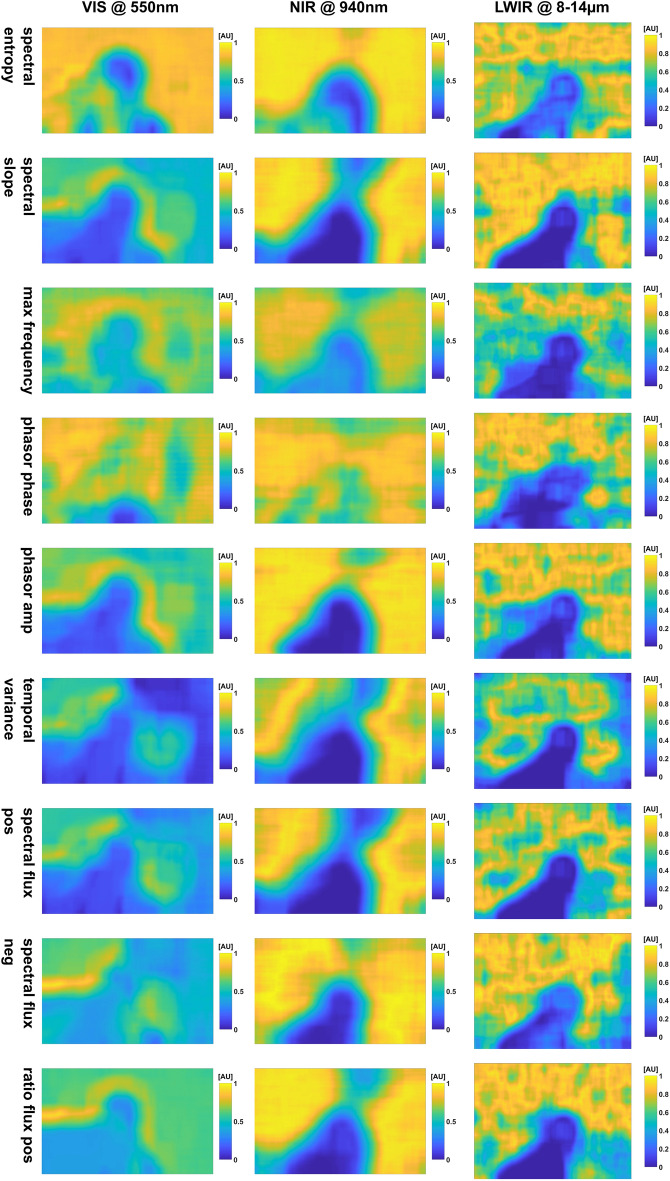
Similarity maps of adult measurement w/ movement: *spectral entropy* to *flux ratios*

**Fig. 26:**
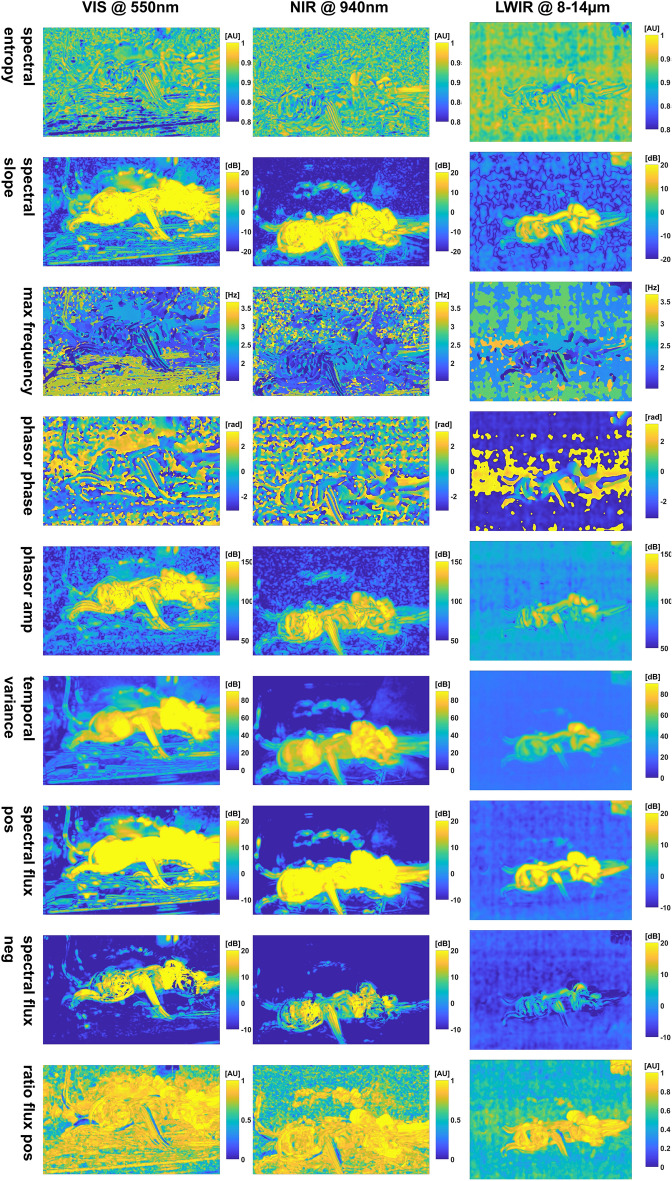
Feature maps of baby measurement w/ movement: *spectral entropy* to *flux ratios*

**Fig. 27:**
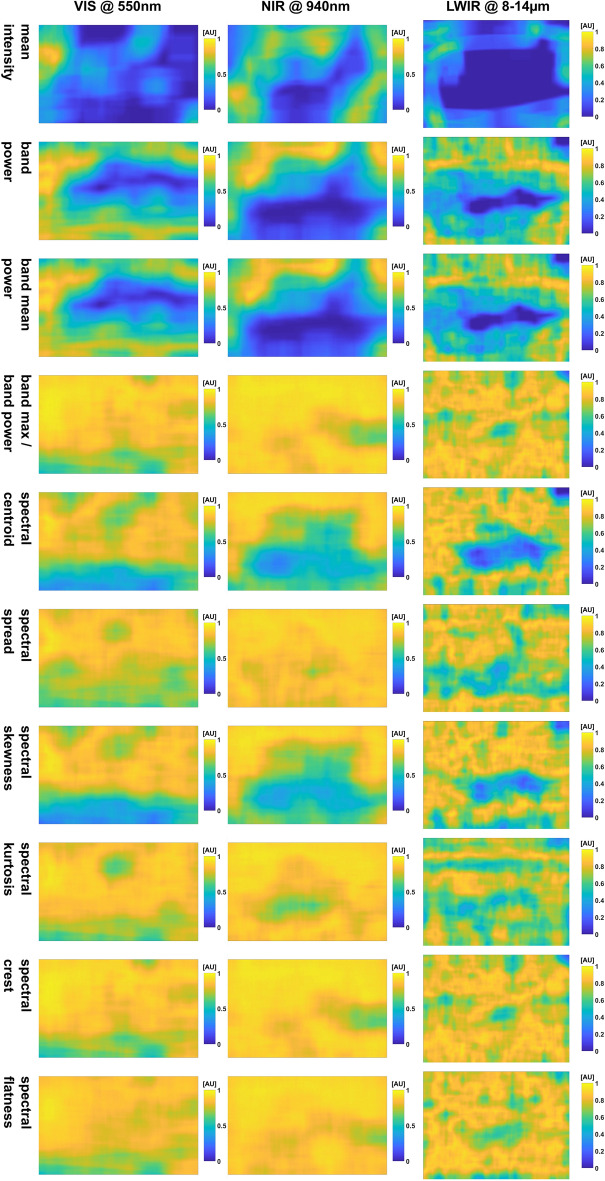
Similarity maps of baby measurement w/ movement: *mean intensity* to *spectral flatness*

**Fig. 28:**
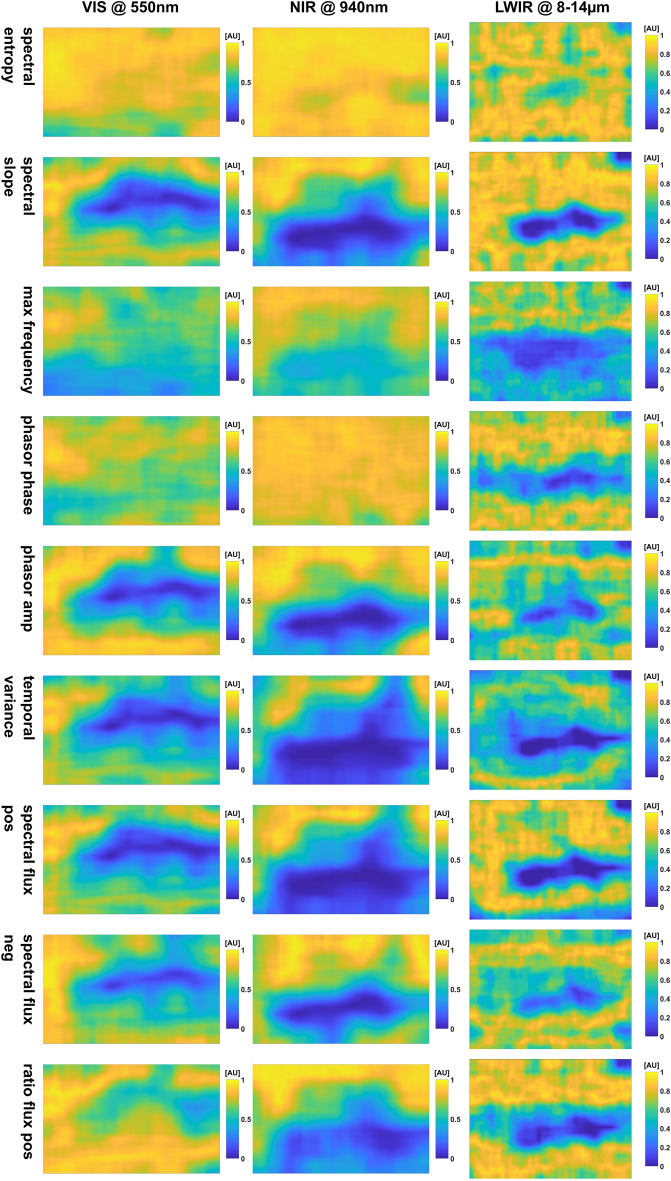
Similarity maps of baby measurement w/ movement: *spectral entropy* to *flux ratios*

## Data Availability

The datasets generated and/or analyzed are not publicly available. However, the adult dataset is available from the corresponding author on reasonable request

## Appendix B: Features

This part of the paper lists the definitions of the features used for the generation of maps as described in Section "Methods".

Features are generated for each segment of a discrete time series $s_{\text {seg},r}[n]$ of a ROI (*r*), where *n* indexes the time. In the following, we omit the index *r* for simplicity. However, spectral features are generated from padded signal segments of the length $N_\text {FFT}$ while the temporal feature uses $N_\text {seg}$ values (Table 3).

### B.1 Temporal features

We use the *mean intensity* image extracted in the temporal processing stage as a visual reference (but not as a feature) (Fig. 2). The mean intensity and temporal (sample) variance are defined for a signal segment as given below:

*Mean intensity*

4 $$\begin{aligned} \mu _\text {intensity,mean}=\frac{1}{N_\text {seg}} \sum _{n=0}^{N_\text {seg}-1}s[n]. \end{aligned}$$

*Temporal (sample) variance*

5 $$\begin{aligned} \mu _\text {variance,sample}={\frac{1}{N_\text {seg}-1}\sum _{n=0}^{N_\text {seg}-1} \left( s[n]-\mu _\text {intensity,mean}\right) ^2}. \end{aligned}$$

### B.2 Spectral features

The calculation of the spectral features uses parts of the one-sided spectra of the DFT and PS. Specifically, a narrow band is defined by a lower and upper frequency bin $b_\text {low}$ and $b_\text {high}$ corresponding to the frequency range selected $f_\text {low,subject}$ and $f_\text {high,subject}$.

We use the PS and calculate the following features using the values at the spectral bins $b_k$ with values $S_k$ and corresponding frequencies $f_k$. The band has $N_\text {band}= (b_\text {high}-b_\text {low}+1)$ spectral bins.

*Band power*

6 $$\begin{aligned} \mu _\text {band}=\sum _{k=b_{ \text {low}}}^{b_{\text {high}}}S_k. \end{aligned}$$

*Band mean power*

7 $$\begin{aligned} \mu _\text {band,mean}=\frac{1}{N_\text {band}}\sum _{k=b_{ \text {low}}}^{b_{\text {high}}}S_k. \end{aligned}$$

*Spectral centroid*

8 $$\begin{aligned} \mu _1=\frac{\sum _{k=b_\text {low}}^{b_\text {high}}{S_kf_k}}{\mu _{\text {band}}}. \end{aligned}$$

*Spectral spread*

9 $$\begin{aligned} \mu _2=\left( \frac{\sum _{k=b_\text {low}}^{b_\text {high}}{{S_k(f}_k-\mu _1)}^2}{\mu _\text {band}}\right) ^\frac{1}{2}. \end{aligned}$$

*Spectral skewness*

10 $$\begin{aligned} \mu _3= \frac{\sum _{k=b_\text {low}}^{b_\text {high}}{{S_k(f}_k-\mu _1)}^3}{{\mu _2}^3\mu _\text {band}} . \end{aligned}$$

*Spectral kurtosis*

11 $$\begin{aligned} \mu _4=\frac{\sum _{k=b_\text {low}}^{b_\text {high}}{{S_k(f}_k-\mu _1)}^4}{{\mu _2}^4\mu _\text {band}}. \end{aligned}$$

*Spectral crest*

12 $$\begin{aligned} \mu _\text {crest}=\frac{\text {max}{\left( S_k\right) }}{\frac{1}{N_\text {band}}\mu _\text {band}},k\in \left[ b_\text {low},b_\text {high}\right] . \end{aligned}$$

*Spectral flatness*

13 $$\begin{aligned} \mu _\text {flatness}=\frac{\left( \prod _{k=b_\text {low}}^{b_\text {high}}S_k\right) ^\frac{1}{N_\text {band}}}{\frac{1}{N_\text {band}}\mu _\text {band}}. \end{aligned}$$

*Ratio of spectral max and band—band max/band power*

14 $$\begin{aligned} \mu _{\mathrm{max, \,tot}}=\frac{\text {max}{\left( S_k\right) }}{\mu _\text {band}},k\in \left[ b_\text {low},b_\text {high}\right] . \end{aligned}$$

*Spectral entropy*

15 $$\begin{aligned} \mu _\text {entropy}=\frac{-\sum _{k=b_\text {low}}^{b_\text {high}}{{S_k\log _2(S}_k)}}{\log _2\left( N_\text {band}\right) }. \end{aligned}$$

*Spectral slope*

16 $$\begin{aligned} \mu _\text {slope}=\frac{\sum _{k=b_\text {low}}^{b_\text {high}}\left( f_k-f_\text {mean}\right) \left( S_k-\mu _\text {band,mean}\right) }{\sum _{k=b_\text {low}}^{b_\text {high}}{{(f}_k-f_\text {mean})}^2}. \end{aligned}$$

*Spectral max frequency*

17 $$\begin{aligned} f_\text {max}=f(k_\text {max})\ \ \text {with}\ \ k_\text {max} = \underset{k}{\arg \max } \left( S_k\right) ,k\in \left[ b_\text {low},b_\text {high}\right] . \end{aligned}$$

#### B.2.1 DFT features

So far, we only have spectral features that use the amplitude, while the phase was not considered.

As we do not know which frequency is the dominating frequency in the band, we calculate the phasor of the whole band and evaluate its phase. Here, we assume that the phasor (which is just a vector) is mainly influenced by the strongest spectral components.

*Phasor*

18 $$\begin{aligned} p_\text {band}=\sum _{k=b_\text {low}}^{b_\text {high}}{S_{k,\text {FFT}}\ \ \text {with}}\ S_{k,\text {FFT}}={\sum _{n=0}^{N_\text {FFT}-1}s \left[ n\right] e}^{-{\frac{i2\pi }{N_\text {FFT}}kn}}.\ \end{aligned}$$

Two features based on phase and or phase and amplitude were computed from the one-sided DFT:

*Phase*

19 $$\begin{aligned} \theta _\text {band}=\text {angle}\left( p_\text {band}\right) . \end{aligned}$$

Values of the phase range between $\pm \pi$. By adding up all phasors to $p_\text {band}$, we hope that in a pulsatile signal the phasors corresponding to the pulsatile component are dominating, while non-pulsatile components cancel each other or have less influence.

*Phasor amplitude*

20 $$\begin{aligned} p_\text {band, amp}= |p_\text {band}|. \end{aligned}$$

#### B.2.2 Features using two time segments

More features are defined by combining two video segments of time *t* and $t-1$. These are used to account for the temporal change of the spectrum and should allow the definition of detectors for onsets and offsets of drastic signal changes, such as movement activity. Conventionally, the spectral flux is defined as the (power) exchange between two segments using a $p-$Norm (e.g., $p=2$ [40]). However, we were interested in how many bins change their values and define positive and negative spectral fluxes and their ratios, respectively:

*Positive and negative spectral flux*

21 $$\begin{aligned} \mu _\text {flux+}= & {} \sum _{k=b_\text {low}}^{b_\text {high}}{S_k\left( t\right) -S_k\left( t-1\right) ,\ \ \text {with}}\ S_k\left( t\right) -S_k\left( t-1\right) \ge 0, \end{aligned}$$

22 $$\begin{aligned} \mu _\text {flux-}= & {} \sum _{k=b_\text {low}}^{b_\text {high}}{S_k\left( t\right) -S_k\left( t-1\right) ,\ \ \text {with}}\ S_k\left( t\right) -S_k\left( t-1\right) <0. \end{aligned}$$

*The ratio of bins with positive/negative flux—ratio flux pos (neg) *

23 $$\begin{aligned} r_\text {flux+}= & {} \frac{1}{N_\text {band}}\sum _{k=b_\text {low}}^{b_\text {high}} B_{+}\left( k\right) , \ \text {with } B_{+}\left( k\right) = {\left\{ \begin{array}{ll} 1 &{}, \text {if } S_k\left( t\right) -S_k\left( t-1\right) \ge 0 \\ 0 &{}, \text {otherwise}. \end{array}\right. } \end{aligned}$$

24 $$\begin{aligned} r_\text {flux-}= & {} \frac{1}{N_\text {band}}\sum _{k=b_\text {low}}^{b_\text {high}} B_{-}\left( k\right) , \ \text {with } B_{-}\left( k\right) = {\left\{ \begin{array}{ll} 1 &{}, \text {if } S_k\left( t\right) -S_k\left( t-1\right) < 0 \\ 0 &{}, \text {otherwise}. \end{array}\right. } \end{aligned}$$

### B.3 Meaning of the features and novelty

As was stated above in Section "Methods", the spectra extracted can be considered as distributions and, therefore, can be assessed by signal statistics. In fact, we need measures to attest the presence of peaks, outliers, etc., of these distributions.

With this in mind, we will briefly discuss some of the features:

The (spectral) kurtosis is often misinterpreted as a measure of ‘peakedness’ of distributions, whereas it is a measure for the weight of the tails and outliers [41].

The (spectral) skewness differentiates between the tails of the distribution: higher frequencies dominate when skewness is negative while lower frequencies are more dominant for positive values. What we observed is that if a sudden (non-rhythmic, percussive) movement is present, the spectrum is less concentrated. Additionally, bins of lower frequencies are more dominant than bins of higher frequencies..

Lower values of the spectral flatness [42] indicate that a spectrum is less flat, and a distinguishable signal might be extractable, while high values indicate noise-like signals. In essence, spectral flatness is the ratio of geometric and arithmetic mean and values range between [0, 1].

Spectral crest and ratio of band max power and band power are very similar to each other: By omitting the normalization, we get a direct measure of the power relations between the band and the maximum. In both cases, the maximum is calculated on the raw spectra without interpolation. The first occurrence of a maximum value defines the spectral bin of the spectrum.

For many of the features, a detailed overview is given by Mathworks [43] and the corresponding functions for generations have been available since the 2019a release of Matlab.

To the best of our knowledge, most of these maps have not been used either for PPGI or IRT. For this reason, we provide an overview. The closest implementations we could find use power maps to define signal-to-noise ratio maps as ratios of the power at a predetermined frequency band centered at a heart rate estimate vs. the power without this narrow band (e.g., [1, 21, 24, 25]) and the average power in the frequency band close to the estimated heart rate [21]. By contrast, our mapping approach requires only the frequency range of the signal anticipated. In fact, many of the maps can be calculated without knowing the exact heart frequency (pulse rate) but might help to select ROIs to determine the frequency in question.

### B.4 Implementation

#### B.4.1 Common terms

Not surprisingly, as can be seen from the equations, several features share the same terms (e.g., $(f_k-\mu _1)$, $\mu _\text {band}$ or the number of bins ($N_\text {band}$) defining the band. When implementing the processing pipeline, these terms should be calculated prior to the features for efficiency.

#### B.4.2 Scaling of DFT components

Please note that the amplitude of the phasor was calculated without scaling all spectral components by 2, whereas the other spectral features have been scaled. However, this factor does not influence the information contained in the map.

#### B.4.3 Processing times

We report the processing times for the feature maps (without scaling for visualization and spatial pooling) on a workstation computer (Intel Xeon E5-1650v3 3.50 GHz, 128GB DDR4-2133) running Matlab 2019b.

We calculated benchmark results for one video segment of the adult NIR video. We computed the times by processing the segment in partitions of up to ten image columns simultaneously (i.e., time series of said columns). The times represent the average times based on one 10-s video segment, i.e., the average for one time series and for the two map resolutions used were calculated and result from multiplication by the total number of signals. The average processing times are shown in Table 5. The cumulative times, i.e., the times including the filtering, transforms, etc., are listed in Table 6. The time for the spatial pooling is not included ($T_\text {pool,PPGI}=35.44\hbox { s}$ and $T_\text {pool,IRT}=4.08\hbox { s}$). The pooling times depend on the spatial resolution and the kernel size $N_\text {kernel}$. The times reported account for the blurring at full resolution. However, it should also be possible to only pool at the subsampling positions to reduce the overhead. All in all, the number of signals and, thus, the spatial resolution is the main factor influencing the time required. Expensive operations are also the signal transformation by the FFT (and PS), filtering and windowing. Noteworthy, but not to be anticipated any differently, are the relatively short processing time of the *temporal variance* and the longer times for the *flux* operations. Furthermore, the processing time for the *spectral skewness* is unexpectedly long in comparison to *spectral kurtosis*. We assume that the implementation in Matlab for calculating the power of a number which can be calculated by repeated squaring is optimized and, thus, explains the time difference between the two very similar features (see (10) and (11)). We also measured the time for the computation of one PPGI similarity map which was $T_\text {similarity,PPG}=14.64\hbox { s}$ (the time for the larger IRT map would consequently be longer). Finally, it must be noted that the parametrization used resulted in processing times that are not suitable for real-time applications.

**Table 5:** Processing times for the maps and a selection of processing steps. The average time based on the calculation of one partitioned video segment is reported

Map/processing step	Time series [$\upmu$s]	Map 376 $\times$ 232 [s]	Map 608 $\times$ 448 [s]
Mean intensity	0.13	0.01	0.03
Band power	0.03	0.00	0.01
Band mean power	0.05	0.00	0.01
Band max/band power	0.16	0.01	0.04
Spectral centroid	0.56	0.05	0.15
Spectral spread	1.37	0.12	0.37
Spectral skewness	4.53	0.39	1.23
Spectral kurtosis	1.82	0.16	0.50
Spectral crest	0.19	0.02	0.05
Spectral flatness	1.39	0.12	0.38
Spectral entropy	1.73	0.15	0.47
Spectral slope	0.98	0.09	0.27
Max frequency	0.11	0.01	0.03
Phasor phase	0.10	0.01	0.03
Phasor amp	0.10	0.01	0.03
Temporal var	5.24	0.46	1.43
Spectral flux pos	1.27	0.11	0.35
Spectral flux neg	1.27	0.11	0.35
Ratio flux pos	1.45	0.13	0.39
Ratio flux neg	1.45	0.13	0.39
*PS*	19.01	1.66	5.18
*Mean subtract*	0.13	0.01	0.04
*Filter*	5.70	0.50	1.55
*Windowing*	1.25	0.11	0.34
*FFT*	10.41	0.91	2.84

**Table 6:** Cumulative processing times for the maps and a selection of processing steps. The average time based on the calculation of one partitioned video segment is reported. Cumulative times are shown, i.e., including preprocessing times of mean subtraction, filtering, windowing, FFT and PS calculation, respectively

Map/processing step	Time series [$\upmu$s]	Map 376$\times$ 232 [s]	Map 608 $\times$ 448 [s]
Mean intensity	0.13	0.01	0.03
Band power	26.26	2.29	7.15
Band mean power	26.27	2.29	7.16
Band max/band power	26.38	2.30	7.19
Spectral centroid	26.79	2.34	7.30
Spectral spread	27.60	2.41	7.52
Spectral skewness	30.75	2.68	8.38
Spectral kurtosis	28.05	2.45	7.64
Spectral crest	26.41	2.30	7.19
Spectral flatness	27.61	2.41	7.52
Spectral entropy	27.95	2.44	7.61
Spectral slope	27.20	2.37	7.41
Max frequency	26.34	2.30	7.17
Phasor phase	17.73	1.55	4.83
Phasor amp	17.72	1.55	4.83
Temporal var	12.46	1.09	3.39
Spectral flux pos	53.72	4.69	14.63
Spectral flux neg	53.72	4.69	14.63
Ratio flux pos	53.90	4.70	14.68
Ratio flux neg	53.90	4.70	14.68
*PS*	19.01	1.66	5.18
*Mean subtract*	0.13	0.01	0.04
*Filter*	5.70	0.50	1.55
*Windowing*	1.25	0.11	0.34
*FFT*	10.41	0.91	2.84

#### B.4.4 Time complexity

We also report the time complexity using the big O notation as the metric. Firstly, we determine the complexity of a feature map. We start by estimating the cost of computing all $S_{k}$ which are present in most features. Computing all $S_{k}$ is of order ${\mathcal {O}}(N_\text {FFT}^2)$ regarding the DFT and DFT-based PS and only of order ${\mathcal {O}}(N_\text {FFT}\log (N_\text {FFT}))$ when using the FFT for both. We assume that the spectra (DFT/FFT and PS) were calculated and all $S_{k}$ are available and can be accessed in constant time ($\in {\mathcal {O}}(1)$). On this assumption, it follows that the equations for the spectral descriptors all have the same linear complexity ($\in {\mathcal {O}}(N_\text {band})$). Computing the mean intensity (4) and temporal variance (5) are only ${\mathcal {O}}(N_\text {seg})$. Filtering a one-dimensional segment is ${\mathcal {O}}(N_\text {seg}N_\text {filterlength} )$. Windowing a segment is ${\mathcal {O}}(N_\text {win})$ which is the same as ${\mathcal {O}}(N_\text {seg})$. Blurring an image is ${\mathcal {O}}(w_\text {kernel}h_\text {kernel}w_\text {image}h_\text {image})$, where *w* and *h* are the width and height of the kernel or image, respectively. Since the Gaussian filter is separable, the complexity can be reduced to ${\mathcal {O}}(w_\text {kernel}w_\text {image}h_\text {image}) + {\mathcal {O}}(h_\text {kernel}w_\text {image}h_\text {image})$.

As an example, the complexity of a map of a spectral feature is given approximately by the following:

25 $$\begin{aligned}&\in \underbrace{ {\mathcal {O}}(w_\text {image}h_\text {image})}_\text {number of ROIs/pixels} \\&\quad \cdot \big [\underbrace{ {\mathcal {O}}(w_\text {kernel}h_\text {kernel}) \cdot {\mathcal {O}}(N_\text {seg}) }_\text {spatial pooling and creation of a time series}+\underbrace{ {\mathcal {O}}(\underbrace{N_\text {seg}}_\text {mean subtraction}+\underbrace{N_\text {seg}N_\text {filterlength}}_\text {temporal filtering}+ \underbrace{N_\text {win}}_\text {windowing} ) }_\text {temporal processing}+ \underbrace{ {\mathcal {O}}(N^2_\text {FFT} +N_\text {band})}_\text {feature generation}\big ]. \end{aligned}$$

This can be further summarized with the big O notation. Thus, (25) turns into:

26 $$\begin{aligned} \in \underbrace{ {\mathcal {O}}(w_\text {image}h_\text {image})}_\text {number of ROIs/pixels} \cdot \big [\underbrace{ {\mathcal {O}}(w_\text {kernel}h_\text {kernel}) \cdot {\mathcal {O}}(N_\text {seg}) }_\text {spatial pooling and creation of a time series}+{ {\mathcal {O}}(\underbrace{N_\text {seg}N_\text {filterlength}}_\text {temporal filtering}) }+ \underbrace{ {\mathcal {O}}(N^2_\text {FFT} )}_\text {DFT/PS}\big ]. \end{aligned}$$

The estimate of the temporal complexity in (26) is therefore consistent with the measured times as given in Table 5.

In the following, the complexity of the similarity maps is determined: Computing a histogram requires accessing all pixels of an image patch defined by the kernel ($\in {\mathcal {O}}(w_\text {kernel, hist}h_\text {kernel, hist})$). For a similarity map, all pixels of a feature map have to be visited ($\in {\mathcal {O}}(w_\text {feature map}h_\text {feature map})$). Computing a model histogram is $\in {\mathcal {O}}(N_\text {model})$, where $N_\text {model}$ is the number of pixels of the model. The histogram intersection (2) is $\in {\mathcal {O}}(N_\text {bins})$ on the basis of the simplified assumption that determining the minimum is $\in {\mathcal {O}}(1)$. Hence, the order of growth of computing a similarity map is:

27 $$\begin{aligned} \in \underbrace{ {\mathcal {O}}(w_\text {feature map}h_\text {feature map})}_\text {number of patches} \cdot \big [ \underbrace{{\mathcal {O}}(w_\text {kernel, hist}h_\text {kernel, hist})}_\text {histogram of an image patch} + \underbrace{{\mathcal {O}}(N_\text {bins})}_\text {intersection}\big ]+\underbrace{{\mathcal {O}}(N_\text {model}}_\text {histogram of a model}). \end{aligned}$$

## Appendix C: Feature and similarity maps

Here, the results for the measurements w/ movement of the subjects are given. In addition, the complete set of feature and similarity maps is provided.

### C.1 Adult measurement

In this section, the feature and similarity maps for the adult measurement (Fig. 11) are provided: Figs. 13, 14, 15 and 16 correspond to the video segment w/o movement, while Figs. 17, 18, 19 and 20 represent a segment w/ movement.

In the following, the results for the measurement w/ movement are presented and discussed.

#### C.1.1 Adult lab measurement w/ movement

This video segment introduces hand to face movement to consume the chili sauce. In addition to the moving arm, the head was also moved. The maps are given in Figs. 17 and 18.

Firstly, we can observe that when strong movements are introduced, more power is available and, consequently many regions tend to low frequencies (see *frequency max*). While many foreground regions can still be observed, the arm is now visible in all mappings (except for *mean intensity*).

The PPGI maps show two new pulsatile formations next to the head. These formations are left and right of the head for the VIS camera and correspond to the light sources left and right of the camera setup. These are caused by the subject’s shadows (we used frontal illumination with one LED brick light left and right of the camera for visible light, while one LED brick was positioned on top of the cameras for NIR illumination). The IRT is not affected as this is a phenomenon caused by illumination.

We can see for all cameras that the upper torso is affected by the arm movement (less visible in NIR due to the FOV). Again, this is well visible due to the increased *flatness* and higher* entropy*. Although the *phase* also shows the subject’s silhouette, an interpretation of the visualization is difficult.

There is more positive than negative *flux* at pixels covering the arm for this time segment, indicating that the newest values taken for the calculation contain the movement. From here on, the more the buffer gets updated, the fewer images belong to the movement and, thus, the trend will shift to negative values.

#### C.1.2 Similarity maps

The similarity maps are given in Figs. 19 and 20.

The results are visually comparable to the case w/o movement presented in the main part of the paper. In addition, when movement is introduced, the dynamic maps of the IRT camera show the subject’s silhouette and not only the contour.

### C.2 Baby measurement

Here, the feature and similarity maps for the baby measurement (Fig. 12) are provided: Figs. 21, 22 , 23 and 24 correspond to the video segment w/o movement, while Figs. 25, 26, 27 and 28 represent a segment w/ movement.

In the following, the results for the measurement w/ movement are presented and discussed.

#### C.2.1 Baby NICU measurement w/ movement

In this scene, the baby moved the head, stretched the legs, and moved the chest and abdomen. The maps are given in Figs. 25 and 26 in the appendix (Section C.2).

The first thing to notice regarding this video segment is that the reflection of the baby in the encasing is more detailed which is in accordance with the higher power due to movement. Moreover, the baby’s body is now visible in the IRT maps. Secondly, most maps show a different texture on the body and surrounding image regions. As a result of this intense movement, the image content of many maps is very hard to grasp even for a human. Positive exceptions are *band power*, *spectral slope*, *phasor amplitude*, *temporal variance* and *spectral flux*.

Amongst other regions, the face was blurred and no facial components could be discerned. Wires outside the body are highlighted by the movement.

#### C.2.2 Similarity maps

Introducing strong movement (Figs. 27 and 28) allows us to distinguish the baby from the background using IRT and ‘decibel’ maps, but also using the *phase*. Similarly, using PPGI, these maps allow a rough determination of the baby’s position.

## Abbreviations

DFT: Discrete Fourier transform
FIFO: First in, first out
FFT: Fast Fourier transform
FOV: Field of view
HD: High definition
IIR: Infinite impulse response
IRT: Infrared thermography
LED: Light-emitting diode
LWIR: Long-wave infrared
NIR: Near-infrared
PPG: Photoplethysmography
PPGI: Photoplethysmography imaging
PS: Power spectrum
RMS: Root mean square
ROI: Region of interest
SD: Standard deviation
STFT: Short-time Fourier transform
VIS: Visible light
